# Differing Dynamics of Intrapersonal and Interpersonal Coordination: Two-finger and Four-Finger Tapping Experiments

**DOI:** 10.1371/journal.pone.0129358

**Published:** 2015-06-12

**Authors:** Kentaro Kodama, Nobuhiro Furuyama, Tetsunari Inamura

**Affiliations:** 1 Kanagawa University, 3-27-1, Rokkakubashi, Kanagawa-ku, Yokohama-shi, Kanagawa-ken, Japan; 2 Waseda University, 2-579-15, Mikajima, Tokorozawa-shi, Saitama-ken Japan; 3 National Institute of Informatics, 2-1-2 Hitotsubashi, Chiyoda-ku, Tokyo, Japan; 4 The Graduate University for Advanced Studies, 2-1-2 Hitotsubashi, Chiyoda-ku, Tokyo, Japan; University of California, Merced, UNITED STATES

## Abstract

Finger-tapping experiments were conducted to examine whether the dynamics of intrapersonal and interpersonal coordination systems can be described equally by the Haken—Kelso—Bunz model, which describes inter-limb coordination dynamics. This article reports the results of finger-tapping experiments conducted in both systems. Two within-subject factors were investigated: the phase mode and the number of fingers. In the intrapersonal experiment (Experiment 1), the participants were asked to tap, paced by a gradually hastening auditory metronome, looking at their fingers moving, using the index finger in the two finger condition, or the index and middle finger in the four-finger condition. In the interpersonal experiment (Experiment 2), pairs of participants performed the task while each participant used the outside hand, tapping with the index finger in the two finger condition, or the index and middle finger in the four-finger condition. Some results did not agree with the HKB model predictions. First, from Experiment 1, no significant difference was observed in the movement stability between the in-phase and anti-phase modes in the two finger condition. Second, from Experiment 2, no significant difference was found in the movement stability between the in-phase and anti-phase mode in the four-finger condition. From these findings, different coordination dynamics were inferred between intrapersonal and interpersonal coordination systems against prediction from the previous studies. Results were discussed according to differences between intrapersonal and interpersonal coordination systems in the availability of perceptual information and the complexity in the interaction between limbs derived from a nested structure.

## Introduction

In daily life, bimanual coordination is an important capability to manipulate an object (e.g., cutting a paper with scissors, having a paper with left hand and a scissor with right hand). Sometimes people also need the ability to communicate with other individuals using gestures or sign language. Such bimanual coordination plays an important role not only for expert pianists in improving their dexterous skills, but also for children during learning or for physically impaired persons in reacquiring some complex finger movement. Rhythmic structures underlie such bimanual coordination [1]. Rhythmic coordinated behaviors can be found not only in bimanual coordination but more generally in our biological activity such as walking or breathing. Which kind of principle does work in such rhythmic coordinated movement?

### Dynamical Systems Approach

Since the first groundbreaking work on inter-limb coordination conducted by Kelso [2], research on this topic has progressed rapidly. Among all reviewed studies, findings obtained using dynamical systems approaches were the following. Although the stability of movement decreased both in the in-phase and anti-phase modes with an increase of movement frequency, bimanual coordination in the in-phase mode is more stable than that in the anti-phase mode at high frequency [3]. An important observation is that phase transitions take place unidirectionally from the anti-phase mode to the in-phase mode when the required oscillation frequency reaches or exceeds a critical point [2]. According to these observations [2], the HKB model was proposed as the first application of the self-organization theory to human movement pattern formation [4]. The HKB model describes the qualitative change (phase transition) of a dynamical system using the concepts of *Synergetics*, a theory of self-organization in non-equilibrium open systems (e.g., an *order parameter* that indexes the macroscopic order or pattern of the system, a *control parameter* that determines the macroscopic state and its spontaneous change of the system [5]). Rhythmic coordinated behaviors such as inter-limb coordination can be modeled as a motion equation using a control parameter and an order parameter [4]. It predicts the behavior of a system, composed of numerous mutually interacting components (degrees of freedom), as the dynamics of few order parameters [4]. In inter-limb coordination, the order parameter is reportedly the relative phase. It describes the low-dimensional behavior (the system’s macroscopic pattern) that arises from the high-dimensional neuromuscular system (the micro components of system). Before the qualitative change, the system fluctuates. Such a loss of stability can be measured by the standard deviation of the relative phase [2, 4]. The HKB model and its framework have been applied to many other movement tasks involving the wrist [6], wrist and elbow [7], forearm [8], and shoulder [9].

### Comparison of Intrapersonal and Interpersonal Coordination Systems

These findings were also obtained for interpersonal coordinated movement such as swinging of the legs [10] or pendulums [11]. The phase transitions in interpersonal coordination systems indicate that visual information underlies the organization of a coordinated movement because these systems involve no mechanical or neural coupling between limbs, which differs from intrapersonal systems. Reportedly for an interpersonal system, the same self-organization principle governs an intrapersonal system as an intrapersonal system, although the coupling strength between limbs is stronger in intrapersonal systems than in interpersonal systems [12, 13]. Recently in the interpersonal coordination paradigm it has been suggested that social factors such as affiliation [14], rapport [15] and context [16] also involve behavioral synchrony or coordination.

Most studies of coordinated movement have examined either intrapersonal or interpersonal coordination of a pair of oscillators (fingers, legs, pendulums, etc.) wiggling or swaying in the air. These studies can elucidate the effects of visual or auditory information, and of neuromuscular coupling in the case of intrapersonal coordination, but not the effect of haptic information in terms of contact on a surface of an environment. Exceptionally, Richardson and his colleagues discussed some haptic feedback related to interpersonal coordination with a rocking chair paradigm. They investigated coordination between people sitting on rocking chairs. They discussed the possibility of shared haptic information between participants in terms of physical vibrations through the ground because, in the case of a rocking chair, they can interact in a haptic manner through the vibration generated by a chair swinging [17].

### Finger-tapping Task

The finger-tapping task requires that participants use not only visual and auditory information, but also haptic information (e.g., looking at a moving finger, listening to auditory metronome stimuli, and touching the desk surface). Reportedly, not only visual information [18] or auditory information [19] but also haptic information [20] can stabilize coordinated movement. It can be said that a tapping task differs in involving haptic information by touching an environmental surface from other wiggling/swinging tasks.

Most previous studies of finger-tapping, including many reviewed in an earlier report [21, 22], however, have been conducted in the sensorimotor synchronization paradigm. In that paradigm, participants were asked to tap a finger unimanually in synchrony with external stimuli such as a metronome beat. Some researchers conducted finger-tapping studies in terms of inter-limb coordination (i.e., bimanual finger-tapping paradigm). Except for polyrhythm studies [23], most researchers investigating bimanual finger-tapping have examined the stability of movement described as a change of particular phase modes (i.e., in-phase and anti-phase) in intrapersonal experiments, with the index finger of each hand, two-finger condition [24,25] or two finger combinations among index, middle, and ring fingers, four-finger condition [26,27]. Although some previous studies have examined bimanual finger-tapping from the perspective of a dynamical systems approach [3], no report in the literature describes a study examining the generality or applicability of the HKB model by comparison of an intrapersonal to an interpersonal coordination system in the finger-tapping paradigm. One interest of the authors is the difference in the effect of haptic information in terms of touching an environmental surface on the dynamics of coordination systems between intrapersonal and interpersonal ones.

Another interest of the authors is the effect of the number of oscillators (i.e., fingers, in the case of tapping task) on coordination dynamics. Although our daily actions using fingers, such as typing at a keyboard or playing piano, require coordination of multiple fingers, no previous report in the literature describes a study that has examined the effects of the number of fingers. Not only for such a practical motivation, but also for a theoretical motivation related to the issues, such as a nested system interacting among components at the different level [28–31], multi-scale interaction [32] and flexibility [3, 33], the present studies were conducted. They did not directly address the emergent property of hierarchal system, its multi-scale interaction or flexibility. Instead, they compare the intrapersonal and interpersonal coordination system as a first step to approach these issues.

### Applicability of the HKB Model

The HKB model and its framework have been applied widely to individual-environment systems (coupling between an agent’s movement and external auditory [34] or visual [35, 36] event), intrapersonal coordination system and interpersonal coordination system. However, some recent reports have described results that throw the model’s generality into question [37–39].

Van Ulzen and colleagues investigated whether the HKB model applies to interpersonal coordination in walking side-by-side on a treadmill. They reported that in-phase and anti-phase mode were equally stable, independent of walking speed and the difference in the individually preferred stride frequencies, and reported that the latter parameter (i.e., detuning term) did not induce systematic phase shift [38]. For a subsequent study, van Ulzen et al. (2010) hypothesized the following:

If the HKB model applies to interpersonal coordination during walking side-by-side, then (1) the variability of in- and antiphase should be minimal, (2) intermediate relative phases should be attracted to either in- or antiphase, and (3) the absolute shift away from the required relative phase should be greatest for 90° phase difference (p.80).

Nevertheless, van Ulzen et al. (2010) reported the results as follows:

(1) relative phase variability was not markedly lower for in- and antiphase coordination, (2) during paced walking in-phase coordination attracted nearby relative phases, whereas antiphase coordination did not, while during unpaced walking both in- and antiphase coordination appeared attractors, and (3) in terms of absolute error, walking at a required relative phase of 90° was indeed the most difficult condition (p.81).

They concluded that these results demonstrate that the HKB model does not apply to interpersonal coordination during gait in a straightforward manner in terms of the HKB hypothesis presented above [38, 39].

These previous studies, however, did not deal with any comparison between intrapersonal and interpersonal coordination, or with any interaction between intrapersonal and interpersonal coordination. It is difficult to investigate how the number of oscillators affects the coordination dynamics using a walking task, because it is difficult for us to walk using one limb in interpersonal coordination or using four limbs in intrapersonal coordination.

### Difference between Intrapersonal and Interpersonal Coordination Systems

Therefore this study relies on the assumption that one difference between intrapersonal and interpersonal coordination systems is the availability of haptic information because the latter system has no neural/mechanical linkage. For the latter system, although each person has access to their own haptic information, no shared haptic information is available. It is also assumed for this study that another difference between intrapersonal and interpersonal coordination system is the complexity of interaction among components related to a nested structure of system. If we can measure only two components interacting, it is difficult to observe a nested system’s behavior. On the other hand, if we can measure four components interacting, it is possible to observe it. In the latter case, a hierarchical system might emerge. Although the present experiment did not examine or analyze the emergent property of hierarchical systems directly, it investigated the effect of number of fingers on the inter-limb coordination dynamics as the first step to address it. To do so, two finger-tapping experiments were conducted, designated respectively as Experiment 1 and Experiment 2 in intrapersonal and interpersonal coordination systems.

For the finger-tapping task, if it is apparent that the general pattern of the in-phase mode becomes more stable than the anti-phase mode over the critical frequency, independent of the number of fingers, then it will support the HKB model's traditional prediction. However, if it is apparent that the in-phase mode and anti-phase mode are equally stable or that the anti-phase mode is more stable than the in-phase, it would indicate the possibility that the HKB model might not be readily applicable (it will not debunk or deny the model). Those factors should be regarded as new terms/parameters in the model if haptic information or the number of oscillators affects the inter-limb coordination dynamics. It might contribute to greater generalization of the model because inter-limb coordination in our daily life such as typing at a keyboard or playing the piano involves touching the environmental surface or/and multi-limb coordination.

This work is building on the wealth of existing literature that describes exploration of intrapersonal and interpersonal coordination dynamics. Within the literature, the current studies investigate the effects of haptic information, in terms of touching the environmental surface, on inter-limb coordination dynamics using a finger-tapping task. The effect of the number of oscillators is also investigated to compare the intra-personal and interpersonal coordination systems differing in terms of hierarchical organization and complexity of interaction among components related to a nested structure of systems.

## Experiment 1: Intrapersonal Tapping Experiment

### Methods

**Fig 1:**
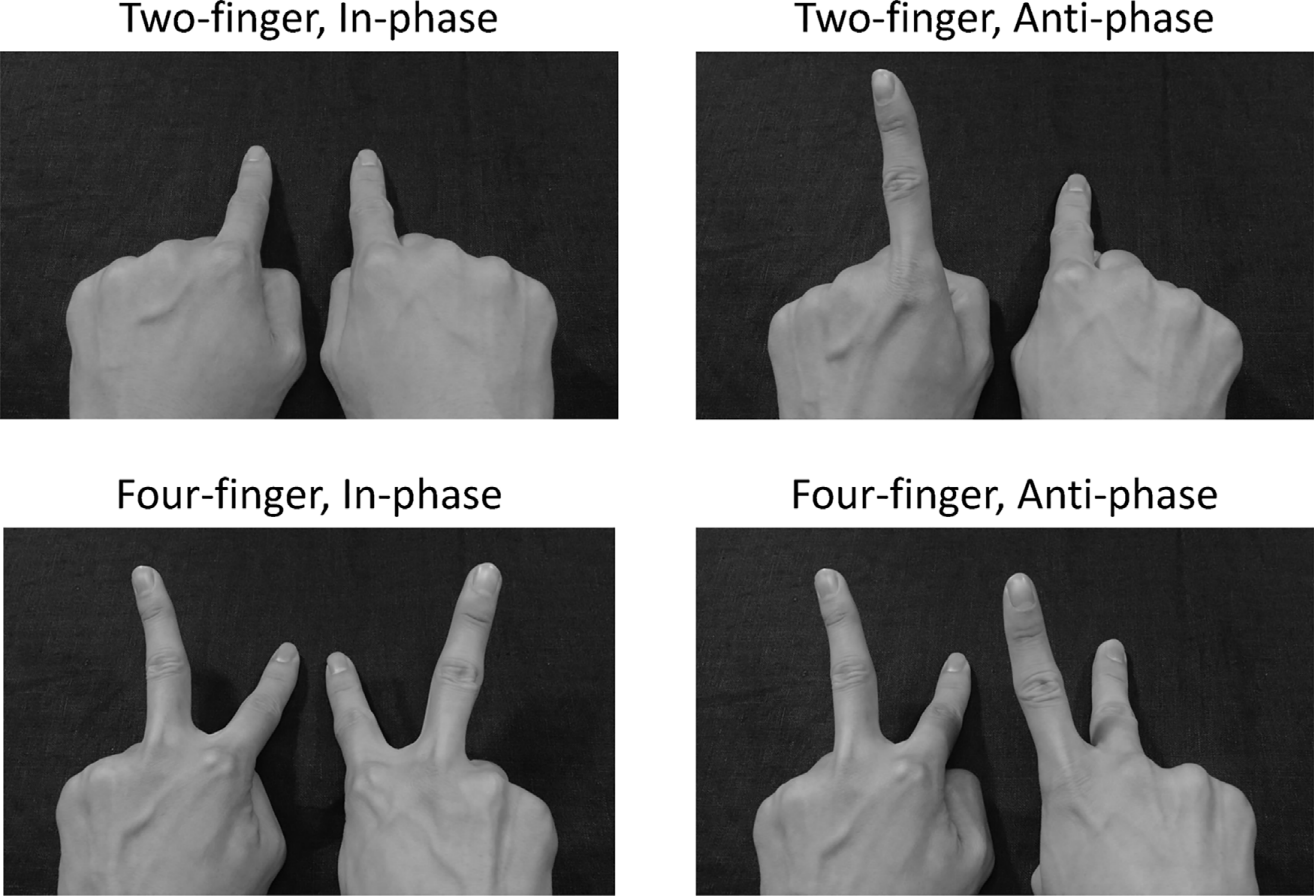
Two within-subject factors. Phase mode: left, in-phase mode; right, anti-phase mode. Number of fingers: top, two-finger; bottom, four-finger.

In Experiment 1 (intrapersonal experiment), two within-subject factors were examined. One factor is the phase mode: in-phase or anti-phase mode (Fig 1: the left two panels show the in-phase condition; the right two panels show the anti-phase condition). In the in-phase condition, two index fingers were tapping in synchrony, but in the anti-phase condition, two index fingers were tapping alternately.

The other factor is the number of fingers: two fingers or four fingers (Fig 1: the upper two panels show the two-finger condition. The lower two panels show the four-finger condition.). Fig 1 shows four conditions in all: the left upper panel presents the two-finger in-phase condition; the upper right panel presents the two-finger anti-phase condition; the lower left panel shows the four-finger in-phase condition; and the lower right panel shows the four-finger anti-phase condition. In the two-finger condition, two index fingers are involved in the task. The four-finger condition involves two index fingers and two middle fingers: four fingers. In the four-finger and in-phase condition, participants were required to tap their index (I) and middle (M) finger in the mode of synchronous tapping of both index fingers in periodic alternation to synchronous tapping of both middle fingers: (_I∙I_), (M_∙_M), and so on. However, in the four-finger and anti-phase condition, participants were required to tap the left middle and the right index finger simultaneously in periodic alternation to synchronous tapping of the left index and the right middle finger: (M_∙I_), (_I∙_M), and so on. The underscore “_” denotes the finger’s extension movement, i.e., the finger is extending not tapping on the desk. The middle dot “∙” denotes separation between left and right hand, i.e., left side of dot means the left hand, right side of dot means the right hand.

### Participants

Ten healthy right-handed participants (5 men, 5 women) participated. Participants were recruited by distributing flyers to advertise the study or by sending e-mail. Participants included undergraduate students of other universities and business people as well as graduate students of the Institute. All participants were 22–27 years of age (average = 25.1). All participants had normal hearing and normal vision. The procedures were approved by the research ethics committee of the National Institute of Informatics, where the experiment was conducted. Each participant provided written informed consent to participate in this study. Each was paid 1,000 JPN yen/hr for their participation.

### Apparatus

**Fig 2:**
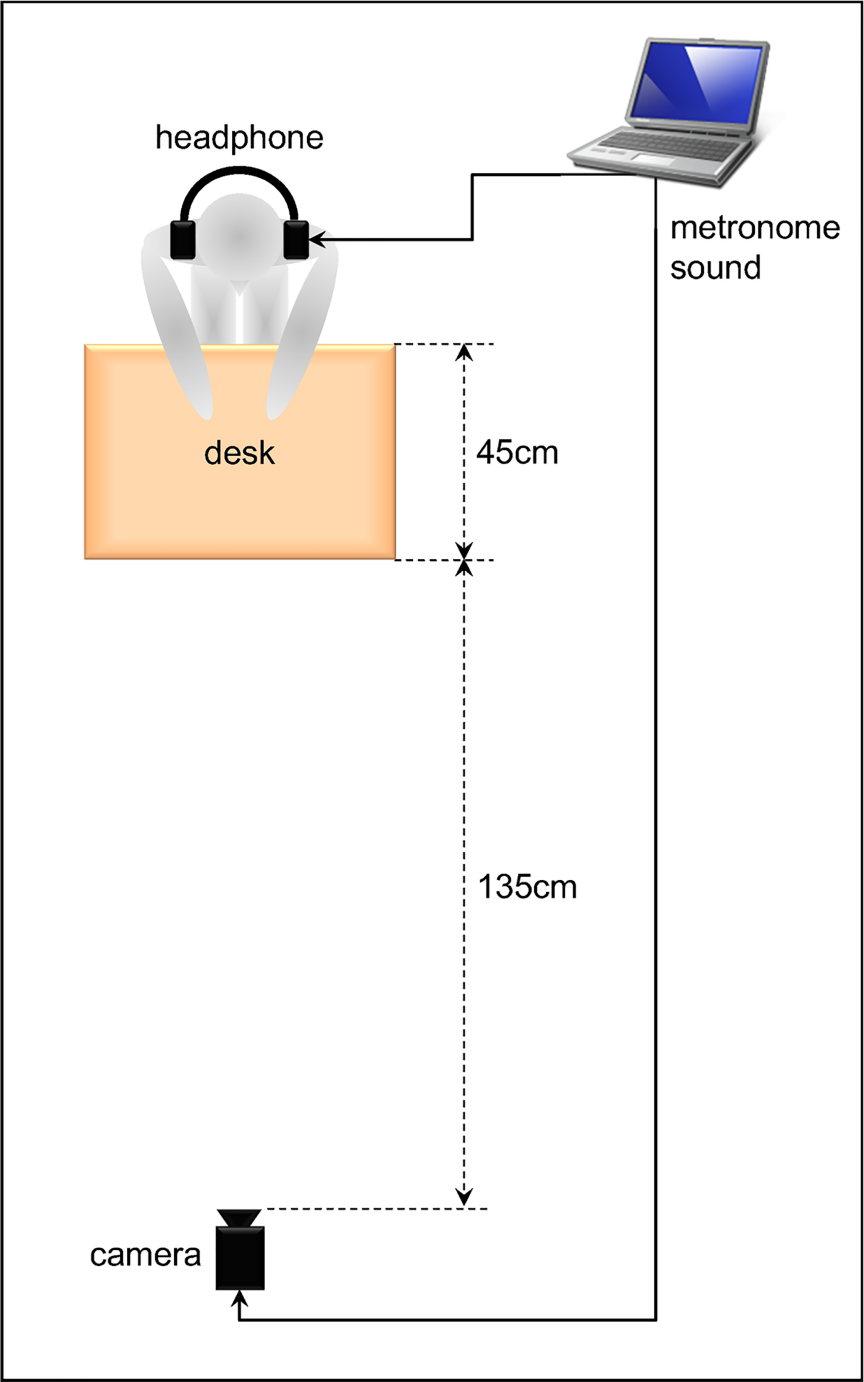
Experimental setup of Experiment 1. Experimental setup of an intrapersonal experiment.

Each participant was seated at a desk in front of a camcorder (TK-C1380; Victor Co. Ltd.) wearing an over-the-ear noise-canceling headphone (MDR-NC600D; Sony Corp.). A computer-generated metronome produced beeps, each lasting 85 ms. The metronome frequency was increased gradually from 1 Hz to 3 Hz over a 30 s trial after an initial 3 s period at 1 Hz. The metronome was run on a personal computer (MacBook2130/13.3; Apple Computer Inc.). The beep sounds were conveyed to participants through headphones at a comfortable volume that was adjusted for each participant. A camcorder, as part of the motion analyzer system (Frame-DIAS II; DKH), videotaped the participants’ index finger movements at 60 fields per second (60 Hz) through the two-dimensional motion capture function of the Frame-DIAS II system. Tapping movements and auditory stimuli were recorded simultaneously on a hard disk drive (HDD). Fig 2 portrays the experimental setup of Experiment 1.

### Design and Procedure

The experiment was designed as a 2 × 2 factorial with two within-subject factors, as shown in Fig 1: phase mode, either in-phase or anti-phase; and the number of fingers, either two-finger or four-finger. Each participant performed tasks in four conditions: two-finger in-phase, two-finger anti-phase, four-finger in-phase and four-finger anti-phase condition. Each condition was repeated four times. The trial order was arranged randomly.

**Fig 3:**
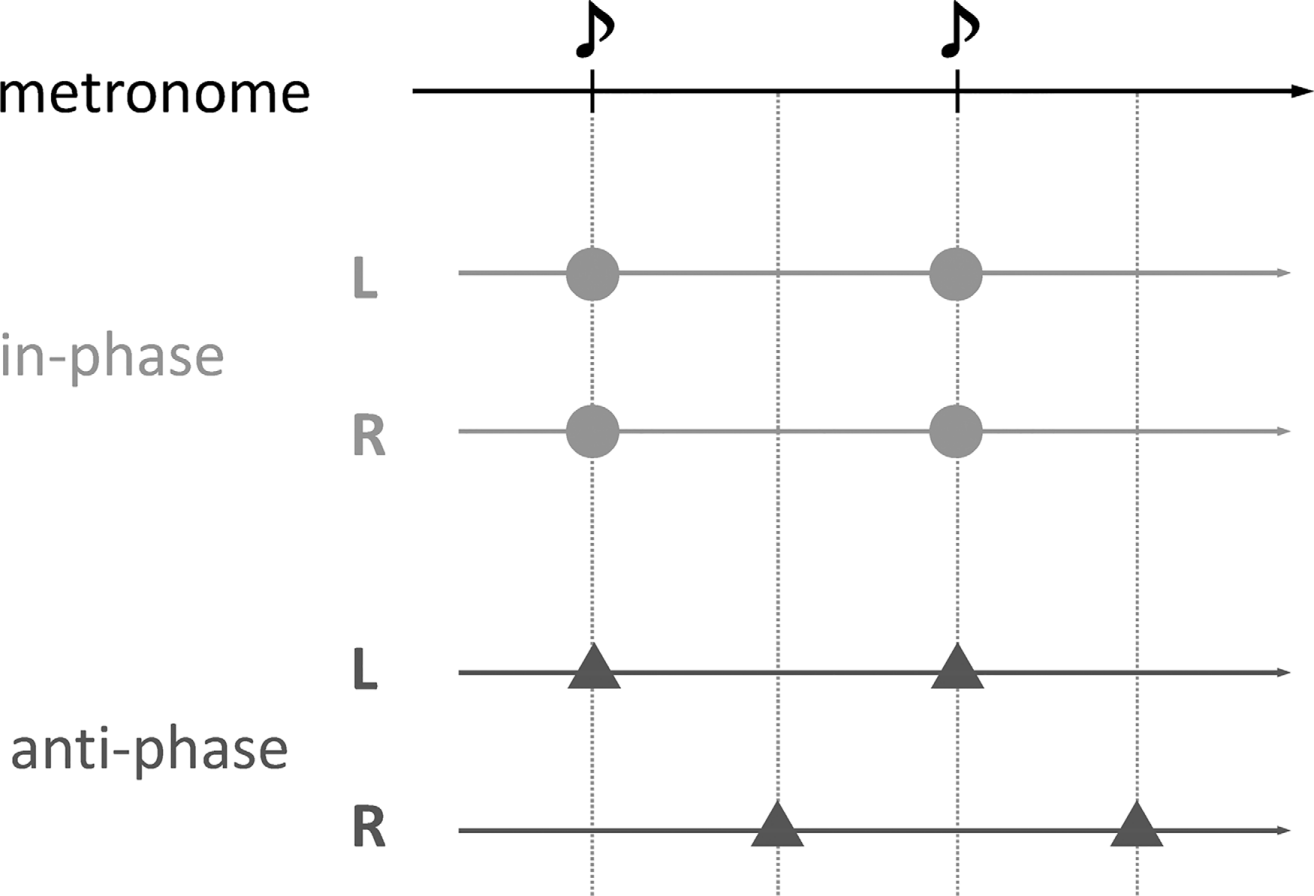
Schematic diagram portraying the temporal relation between taps and beats. L, left hand tap; R, right hand tap; ♪, metronome beat; Time elapses from left to right.

The task was to tap either in the in-phase mode (two index fingers tapping in synchrony) or in the anti-phase mode (two index fingers tapping alternately) at a pace dictated by the auditory metronome: it increased gradually from 1 Hz to 3 Hz over a 30 s trial. Participants were instructed to keep their eyes open, to watch their tapping movements during a trial, and to complete one full movement cycle, an extension-flexion cycle, for each beat of the metronome. They were also instructed to maintain the initial mode of coordination to the greatest degree possible, but not at the expense of losing pace with the metronome. They were told not to resist if they felt a change in the coordination pattern as a result of the increased tapping frequency, to prevent the effect of participants’ intention or effort such as “not to change the pattern” and observe just a natural spontaneous behavior, as in several previous studies [26, 27, 34]. Fig 3 portrays a schematic diagram showing the temporal relation between taps and metronome beats.

### Data Analysis

To assess the tapping movement stability, we show the percentage of phase transition occurrence for each condition. This index, however, revealed only the total stability across a trial (i.e., how often a phase transition occurred). Furthermore, we analyzed the relative phase quantitatively through a trial to assess the frequency effect: how progressively the movement stability increased or decreased. To investigate the relation between the occurrence of phase transition and the movement frequency, the movement frequency was calculated. Additionally a nonlinear analysis method for two time series, cross recurrence quantification analysis was performed.

#### Percentage of phase transition occurrence

The percentage of phase transition occurrence was calculated as follows. First, the phase range threshold regarded as in-/anti-phase mode was defined by analyzing the relative phase (as a result, we judged ±50° as appropriate, which means that 0±50°as in-phase mode and 180±50° as anti-phase mode, because the relative phase reaches 50° at a maximum even under the condition in which no phase transition occurred). Second, the number of times the taps in the opposite phase mode (i.e., in the in-phase condition the opposite phase mode is the anti-phase, vice versa) repeated was referred from results of a preliminary study. Our preliminary study examined when the actor felt “the pattern had changed”. If the actor repeats the taps in the opposite phase mode two times, it means one cycle. We inferred that two or three cycle repetitions in the opposite phase mode can occur by chance. Actually the actors in the preliminary study reported they felt the change when four cycle (i.e., five times) repetition occurred. Then we judged five times as appropriate. Finally, the percentage of phase transition occurrence for each condition was calculated by counting the repetitions of taps in the opposite phase mode. The five-tap cutoff was motivated a priori by result of a previous study, but we also analyzed how the percentages change depending on the number (2, 3, and 4 times) to examine the data carefully.

#### Relative Phase Analysis

The tap times, when the reference finger and target finger tapped the desk (*t*_*Ref*_ and *t*_*Tar*_ respectively denoting the tap times of reference and target fingers), were calculated. Next, the discrete relative phase (φ) between taps was calculated following the procedures described in reports of previous studies [10, 18, 40], based on tap intervals according to the following formula.

$$\varphi =\frac{t_{Tar,i}-t_{Ref,\;i}}{t_{Ref,i+1}-t_{Ref,\;i}}\times 360\;\left[\text{deg}\right]$$

Relative phases were calculated between index fingers (*φ*_*I*_) in the two-finger condition, and additionally between middle fingers (*φ*_*M*_), within the left hand (*φ*_*L*_) and the right hand (*φ*_*R*_) in the four-finger condition. For each combination of fingers above, the discrete point estimates of the relative phase between taps were calculated: *t* *_Ref,i+1_* is the time of reference finger of i+1th tap, *t*_*Ref*,*i*_ is the time of reference finger of *i*-th tap, and *t*_*Tar*,*i*_ is the time of target finger of the *i*-th tap.

To assess the effects of frequency on the stability of finger-tapping movement in this study, each trial was separated into six equal time intervals of 5 s (a 30 s trial was divided into six frequency ranges consisting of 5 s duration) [27]. The standard deviation of the relative phase (SD φ) was calculated for each time interval. For comparing the two-finger and four-finger conditions, φ was calculated between two index fingers (*φ*_*I*_), as in a previous study [27]. Here φ is regarded as an *order parameter*, which can be regarded as an index of the order of movements. The movement frequency is regarded as a *control parameter*, which determines the qualitative pattern change in the order parameter. Here, SD φ is regarded as an index of the movement stability: larger SD φ signifies less stability.

#### Movement Frequency

The movement frequency of the participants was calculated to investigate the relation between the occurrence of phase transition and the movement frequency because it is possible that transitions occur because participants cannot follow the fast metronome frequencies. To deny such a possibility, all trials were sorted into two groups by whether transition occurred or not. Then the maximum frequency in each trial was calculated and averaged across each group. These values were compared statistically between two groups by *t*-test.

#### Cross Recurrence Quantification Analysis

We also conducted cross recurrence quantification analysis (CRQA) [41] on two time series of finger movement. It is a nonlinear method that captures the recurring properties and patterns of a dynamical system, which results from two streams of information interacting over time [41, 42], and quantifies how similarly two observed data series unfold over time [43]. Recurrence quantification analysis was originally developed to uncover subtle time correlations and repetitions of patterns, and is relatively free of assumption about data size and distribution [44]. In CRQA, two time-delayed copies of the original time series were used for embedding the data in higher dimensional space, reconstructing the phase space of the dynamical system, to analyze recurrent structure between them [41]. For inter-limb rhythmic coordination, two CRQA measures are regarded as significant indexes of the movement stability [45, 14]. The percent recurrence (%*REC*) in CRQA corresponds to the ratio of the number of shared locations relative to the number of possible shared locations in phase space. It provides an index of the magnitude of noise in the system [45]; higher %*REC* indexes lower noise in the system. The other is related to the line structure calculated from the recurrence plot (e.g., *Maxline* is the longest shared trajectory in phase space and the length of maximum diagonal line on the plot) [46]. It is a measure of the stability of the shared activity [43]. It provides an index of the system’s sensitivity to perturbations (i.e., the strength of the attractor against perturbations) [45]. The present study calculated the average of the diagonal line (*L*) [42] as a measure of the movement stability because Shapiro-Wilk normality test revealed *Maxline* did not have the normal distribution (W = .9033, *p*<.001).

We performed CRQA using the R package '*crqa*' (version 1.0.5) [42] after determining the optimal values for the input parameters (e.g., *time delay*, *embedding dimensions*, *radius*) using the package [42] and MATLAB toolbox 'CROSS RECURRENCE PLOT TOOLBOX' (version 5.17) [47] and referring the standard guidelines of RQA method [46]. As a result, we chose *time delay* values that correspond to one quarter of a cycle of each movement frequency range, i.e., 0–5 s (mean 1.06 Hz), 5–10 s (mean 1.23 Hz), 10–15 s (mean 1.50 Hz), 15–20 s (mean 1.93 Hz), 20–25 s (mean 2.55 Hz), 25–30 s (mean 2.96 Hz), five *embedding dimensions*, and 0.84 Euclidean distance (*radius*) in phase space.

## Results

### Coordination between index fingers

#### Percentage of phase transition occurrence

**Fig 4:**
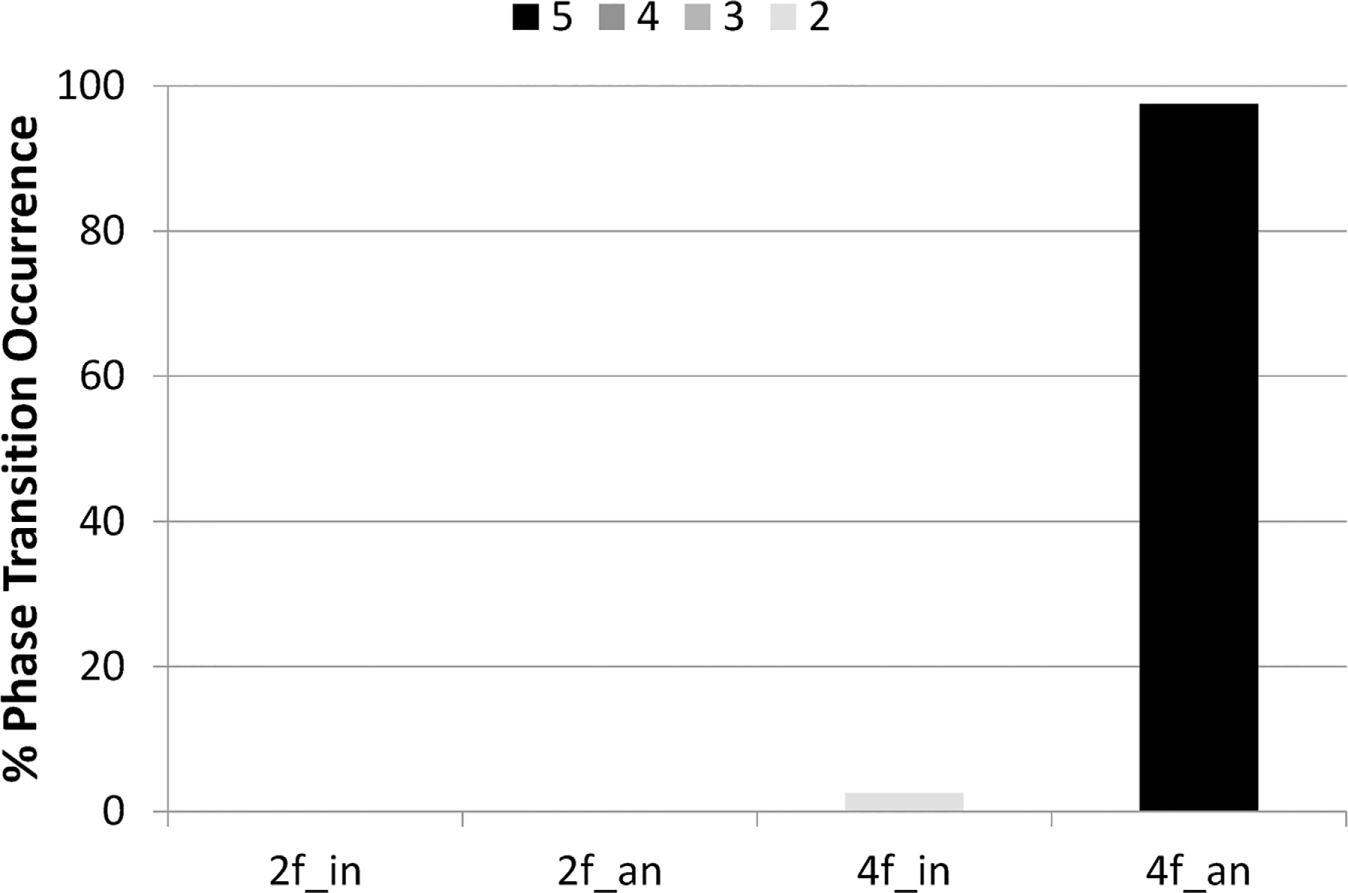
Percentage of phase transition occurrence. Colors of bar indicate how many times the taps in the opposite phase mode repeated. From black (5 times), … to the lightest gray (2 times). 2f-in: two-finger in-phase condition, 2f-an: two-finger anti-phase condition, 4f-in: four-finger in-phase condition, 4f-an: four-finger anti-phase condition.

Fig 4 presents the percentage of phase transition occurrence for each condition. In the two-finger condition, no transition was observed in the in-phase or anti-phase condition. For the four-finger condition, no transition was observed in the in-phase condition, but the transition occurred at 97.5% in the anti-phase condition. In summary, in the two-finger condition, no difference in the percentage of phase transition occurrence was found between two phase modes, whereas in the four-finger condition, the transition occurred more often in the anti-phase condition than in the in-phase condition. No transition was observed in the three conditions except for the four-finger anti-phase condition. Therefore, we conducted no statistical analysis of the percentage of phase transition occurrence for avoiding the flooring effect, i.e., the data without four fingers anti-phase condition were zero, and have no variance. Fig 4 also shows that the percentage of phase transition occurrence was robust even though the parameter, the number of times the taps in the opposite phase mode, changed from 2 to 5.

#### SD of relative phase

**Fig 5:**
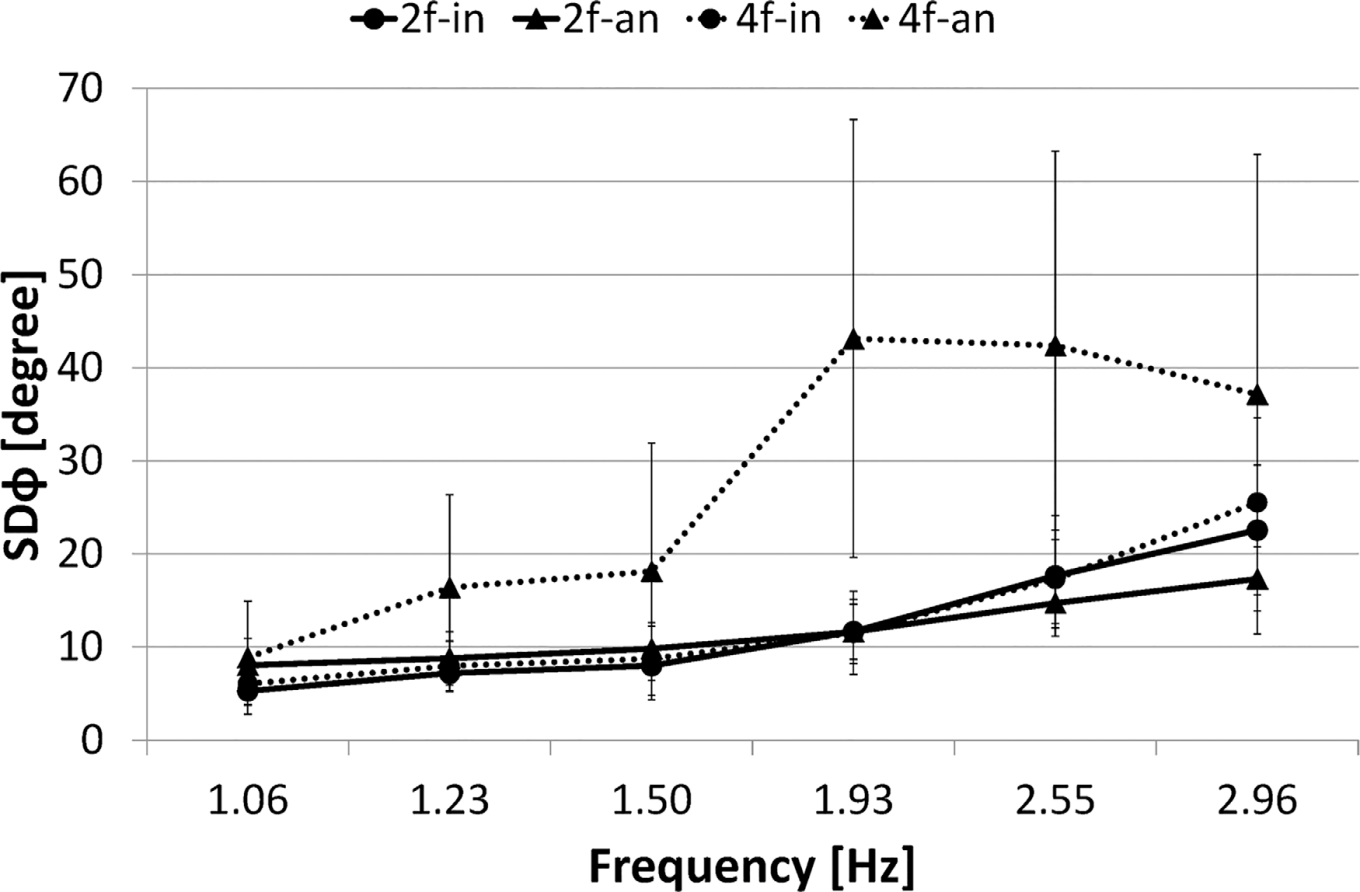
SD of relative phase. Circle marker/rigid line represents two-finger in-phase condition, 2f-in; Triangle marker/rigid line represents two-finger anti-phase condition, 2f-an; Circle marker/dashed line represents four-finger in-phase condition, 4f-in; Triangle marker/dashed line represents four-finger anti-phase condition, 4f-an. Error bars represent the standard deviation.

Fig 5 presents the standard deviation of the relative phase between index fingers (SD *φ*_*I*_) as a function of the movement frequency (mean frequency was calculated, respectively, across each 5 s duration: 1.06, 1.23, 1.50, 1.93, 2.55, and 2.96 Hz).

A three-way ANOVA (number of fingers (2) × phase mode (2) × frequency (6)) conducted on the SD *φ*_*I*_ confirmed the main effect of number of fingers (F(1,9) = 39.983, *p*< .001), phase mode (F(1,9) = 31.530, *p*< .001), and frequency (F(1,9) = 24.500, *p*< .001). It also revealed significant interactions: number of fingers × phase mode (F(1,9) = 44.699, *p*< .001), number of fingers × frequency (F(1,9) = 4.010, *p*< .005), phase mode × frequency (F(1,9) = 2.994, *p*< .05) and number of fingers × phase mode × frequency (F(1,9) = 4.764, *p*< .005).

The simple main effect test for number of fingers × phase mode interaction revealed significant difference in the anti-phase condition between the two-finger and four-finger conditions (F(1,9) = 83.749, *p*< .001), and in the four-finger condition between the in-phase and anti-phase conditions (F(1,9) = 74.505, *p*< .001). However, it revealed no significant difference in the in-phase condition between the two-finger and four-finger conditions (F(1,9) = 0.207, *p* = .6548, N.S.), or in the two-finger condition between the in-phase and anti-phase conditions (F(1,9) = 0.039, *p* = .8452, N.S.). In summary, SD *φ*_*I*_ was significantly larger in the four-finger anti-phase condition than in any of the other three conditions. On the other hand, SD *φ*_*I*_ did not significantly differ between two phase modes in the two-finger condition.

The simple main effect test for number of fingers × frequency interaction revealed significant difference in the high frequency ranges 15–20, 20–25, and 25–30 s between the two-finger and four-finger conditions (F(1,9) = 26.564, *p*< .001, F(1,9) = 20.169, *p*< .001, F(1,9) = 14.066, *p*< .001, respectively). However, it revealed no significant difference in the low frequency ranges (0–5, 5–10, 10–15 s) between the two-finger and four-finger conditions. In summary, SD *φ*_*I*_ was significantly larger in the four-finger condition than in the two-finger condition in the high frequency range.

The simple main effect test for phase mode × frequency interaction revealed significant difference in the frequency ranges 15–20 and 20–25 s between the in-phase and anti-phase conditions (F(1,9) = 27.676, *p*< .001, F(1,9) = 13.625, *p*< .001, respectively), but no significant difference in other frequency ranges between two phase modes. SD *φ*_*I*_ was significantly larger in the anti-phase condition than in the in-phase condition in the specific frequency ranges (15–25 s).

The simple effect test for number of fingers × phase mode × frequency interaction revealed significant simple-simple main effect in the four-finger in the frequency ranges 5–10, 10–15, 15–20, 20–25, and 25–30 s, and significant difference between the in-phase and anti-phase conditions (F(1,9) = 4.118, *p*< .05, F(1,9) = 5.105, *p*< .05, F(1,9) = 57.957, *p*< .001, F(1,9) = 36.503, *p*< .001, F(1,9) = 7.793, *p*< .01, respectively). No significant difference in the two-finger condition was found in any frequency range between the in-phase and anti-phase conditions. In summary, although SD *φ*_*I*_ was significantly larger in the in-phase condition than in the anti-phase condition in the four-finger condition, it did not differ significantly between two phase modes in the two-finger condition over a trial.

#### Cross recurrence quantification analysis

**Fig 6:**
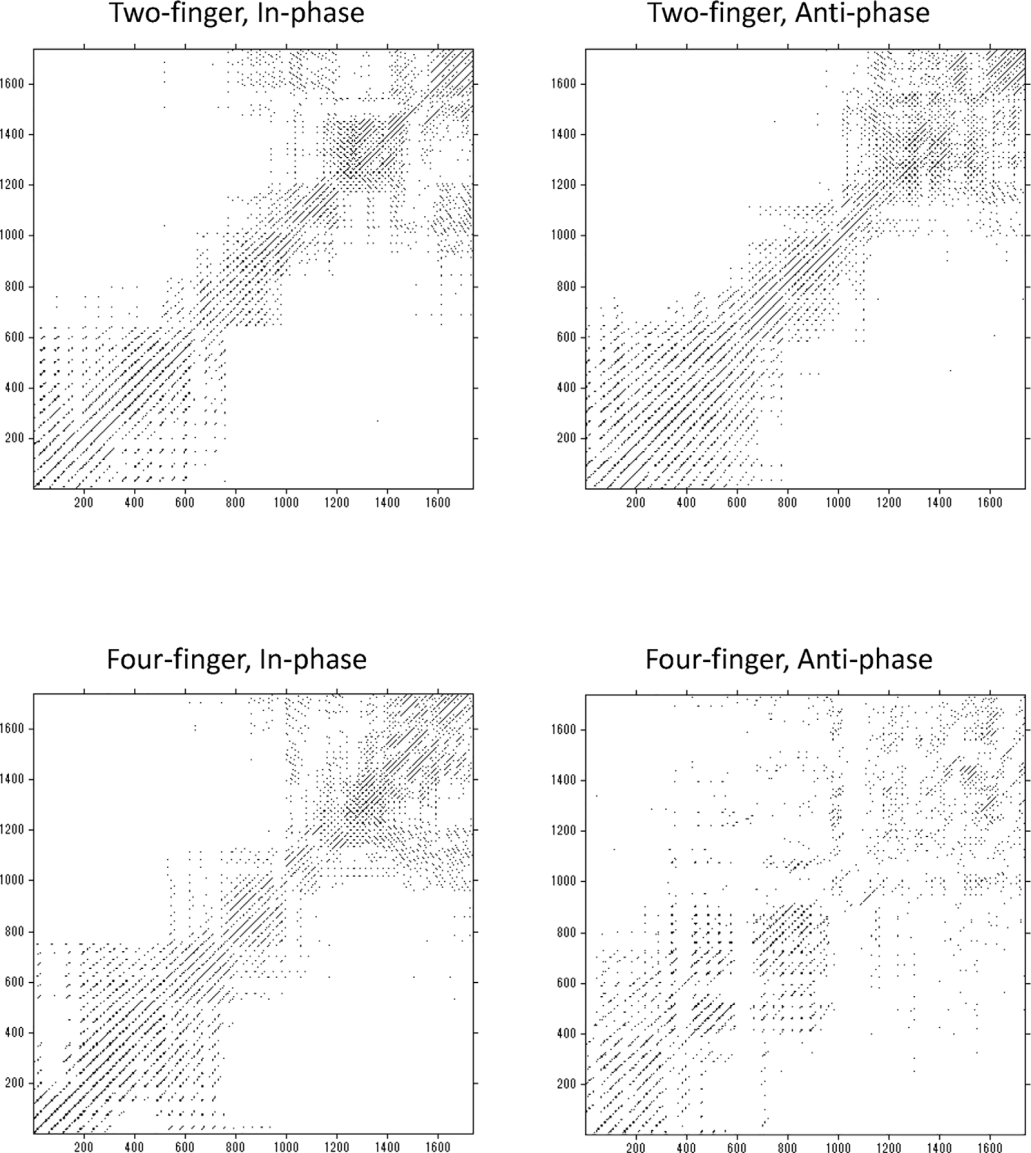
Cross recurrence plots. Cross recurrence plots of sample data for each of four conditions. Left-top, two-finger in-phase condition; Right-top, two-finger anti-phase condition; Left-bottom, four-finger in-phase condition; Right-bottom, four-finger anti-phase condition.

**Fig 7:**
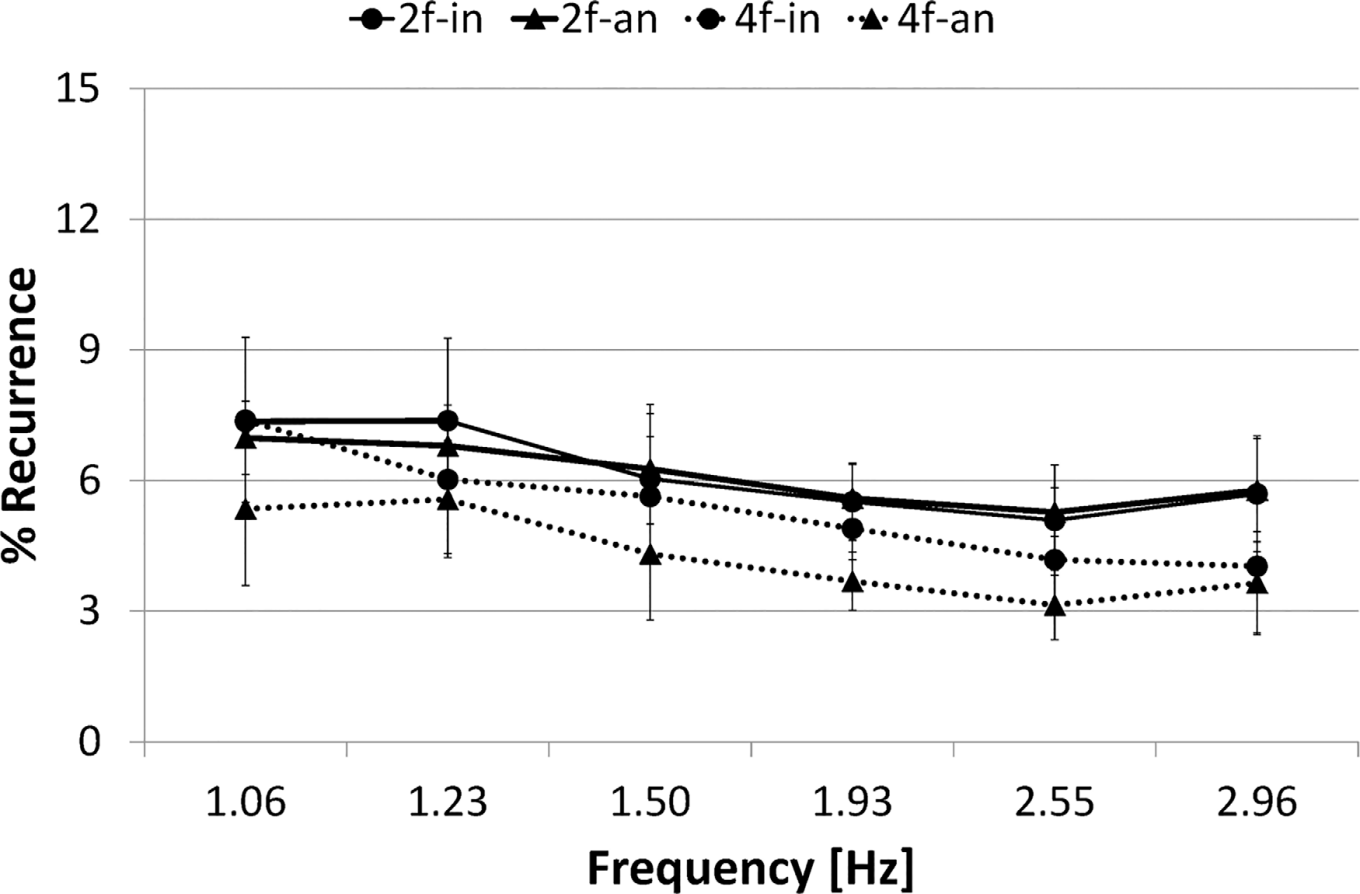
%Recurrence. Circle marker/rigid line represents two-finger in-phase condition, 2f-in; Triangle marker/rigid line represents two-finger anti-phase condition, 2f-an; Circle marker/dashed line represents four-finger in-phase condition, 4f-in; Triangle marker/dashed line represents four-finger anti-phase condition, 4f-an. Error bars represent the standard deviation.

Fig 6 represents cross recurrence plots of sample data for each of four conditions and Fig 7 presents %*REC*_*I*_ for each condition as a function of frequency.

A three-way ANOVA (number of fingers (2) × phase mode (2) × frequency (6)) conducted on %*REC*_*I*_ confirmed the main effect of number of fingers (F(1,9) = 50.650, *p*< .001) and frequency (F(1,9) = 19.292, *p*< .001). It also revealed significant interaction: number of fingers × phase mode (F(1,9) = 29.304, *p*< .001).

The simple main effect test for number of fingers × phase mode interaction revealed significant difference in the in-phase condition between the two-finger and four-finger conditions (F(1,9) = 15.466, *p*< .001), in the anti-phase condition between the two-finger and four-finger conditions (F(1,9) = 77.245, *p*< .001), and in the four-finger condition between the in-phase and anti-phase conditions (F(1,9) = 12.358, *p*< .005). However, it revealed no significant difference in the two-finger condition between the in-phase and anti-phase conditions (F(1,9) = 0.044, *p* = .8364, N.S.). In summary, no significant difference was found in%*REC*_*I*_ between two phase modes in the two-finger condition.

**Fig 8:**
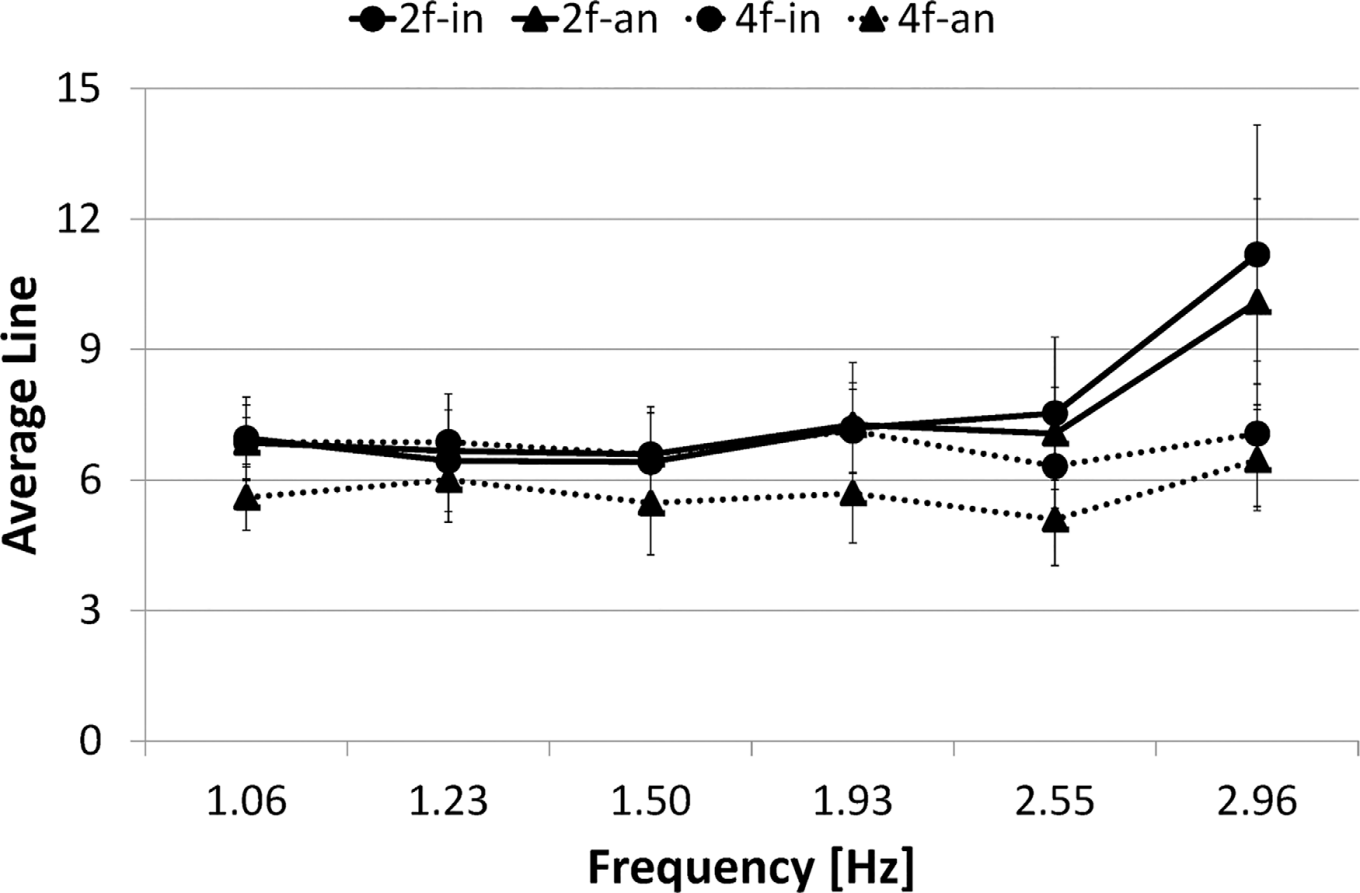
Average line length. Circle marker/rigid line represents two-finger in-phase condition, 2f-in; Triangle marker/rigid line represents two-finger anti-phase condition, 2f-an; Circle marker/dashed line represents four-finger in-phase condition, 4f-in; Triangle marker/dashed line represents four-finger anti-phase condition, 4f-an. Error bars represent the standard deviation.

Fig 8 presents *L*_*I*_ for each condition as a function of frequency. A three-way ANOVA (number of fingers (2) × phase mode (2) × frequency (6)) conducted on *L*_*I*_ confirmed the main effect of number of fingers (F(1,9) = 32.795, *p*< .001) and frequency (F(1,9) = 15.629, *p*< .001). It also revealed significant interactions: number of fingers × phase mode (F(1,9) = 17.071, *p*< .005) and number of fingers × frequency (F(1,9) = 22.627, *p*< .001).

The simple main effect test for number of fingers × phase mode interaction revealed significant difference in the in-phase condition between the two-finger and four-finger conditions (F(1,9) = 11.139, *p*< .005), in the anti-phase condition between the two-finger and four-finger conditions (F(1,9) = 48.413, *p*< .001), and in the four-finger condition between the in-phase and anti-phase conditions (F(1,9) = 12.270, *p*< .005). However, it revealed no significant difference in the two-finger condition between the in-phase and anti-phase conditions (F(1,9) = 0.410, *p* = .5299, N.S.). In summary, no significant difference was found in *L*_*I*_ between two phase modes in the two-finger condition.

The simple main effect test for number of fingers × frequency interaction revealed significant difference in the frequency ranges 15–20, 20–25 and 25–30 s between the in-phase and anti-phase conditions (F(1,9) = 5.678, *p*< .05, F(1,9) = 21.518, *p*< .001, F(1,9) = 127.705, *p*< .001, respectively). In summary, *L*_*I*_ was longer in the two-finger condition than in the four-finger condition in the higher frequency ranges 15–30 s, although no significant difference in *L*_*I*_ was found between them in the lower frequency ranges 0–15 s.

As a result of analyzing the movement frequency, it was revealed that the average maximum frequency across trials in which the phase transition occurred was 2.967 Hz (SD = 0.236), and the average maximum frequency across trials in which no phase transition occurred was 2.945 Hz (SD = 0.303). Results of Welch’s *t*-test indicated no significant difference between the average maximum frequencies of two groups (i.e., the phase transition group and the no phase transition group) (*t*(54) = 0.417, *p* = .678, N.S.).

### Additional analyses of four-finger condition

In the four-finger condition, additional analyses to investigate coordination among four finger combinations including not only between-hand coordination (between index fingers and middle fingers of both hands) but also within-hand coordination (between index and middle fingers of left and right hand). The standard deviation of the relative phase between each finger combination (index fingers *φ*_*I*_, middle fingers *φ*_*M*_, index-middle fingers of left hand *φ*_*L*_, index-middle fingers of right hand *φ*_*R*_) were calculated. CRQA was also conducted on four finger combinations, and two measures (%*REC* and *L*) were obtained for each finger combination (%*REC*_*I*_, *L*_*I*_, %*REC*_*M*_, *L*_*M*_, %*REC*_*L*_, *L*_*L*_, %*REC*_*R*_, *L*_*R*_).

#### SD of relative phase

A three-way ANOVA (finger combination (4) × phase mode (2) × frequency (6)) conducted on the SD of four finger combinations (i.e., *φ*_*I*_, *φ*_*M*_, *φ*_*L*_, *φ*_*R*_) confirmed the main effect of the finger combination (F(1,9) = 4.379, *p*< .05), phase mode (F(1,9) = 36.863, *p*< .001), and frequency (F(1,9) = 41.117, *p*< .001). It also revealed significant interactions: finger combination × phase mode (F(1,9) = 20.864, *p*< .001), finger combination × frequency (F(1,9) = 3.066, *p*< .001), phase mode × frequency (F(1,9) = 5.050, *p*< .001), and finger combination × phase mode × frequency (F(1,9) = 2.728, *p*< .005).

As a result of multiple comparisons using Ryan’s method [48] in the main effect of finger combination, significant difference was found between *φ*_*M*_ and *φ*_*R*_ (t(27) = 3.442, *p*< .005) and between *φ*_*I*_ and *φ*_*R*_ (t(27) = 2.648, *p*< .05). SD φ was smaller within the right hand (*φ*_*R*_) than between hands (*φ*_*I*_ and *φ*_*M*_).

The simple main effect test for finger combination × phase mode interaction revealed significant difference in *φ*_*I*_ and *φ*_*M*_ between the in-phase and anti-phase conditions (F(1,9) = 68.310, *p*< .001, F(1,9) = 44.601, *p*< .001, respectively). However, it revealed no significant difference in *φ*_*L*_ and *φ*_*R*_ between the in-phase and anti-phase conditions (F(1,9) = 0.610, *p* = .4397, N.S., F(1,9) = 3.010, *p* = .0913, N.S., respectively). In summary, SD φ was significantly larger in the anti-phase condition than in the in-phase condition in between-hand combination (*φ*_*I*_ and *φ*_*M*_), but no significant difference between two phase modes in within-hand combination (*φ*_*L*_ and *φ*_*R*_).

The simple main effect test for finger combination × frequency interaction revealed significant difference in 15–20, 20–25 and 25–30 s among finger combinations (F(1,9) = 7.343, *p*< .001, F(1,9) = 3.910, *p*< .01, F(1,9) = 5.984, *p*< .001, respectively). SD φ varied among finger combinations in the high frequency ranges, but no common pattern of the differences was found.

The simple main effect test for phase mode × frequency interaction revealed significant difference in the high frequency ranges 10–15, 15–20, 20–25 and 25–30 s) between the in-phase and anti-phase conditions (F(1,9) = 5.325, *p*< .05, F(1,9) = 24.941, *p*< .001, F(1,9) = 34.732, *p*< .001, F(1,9) = 19.277, *p*< .001, respectively). SD φ was significantly larger in the anti-phase condition than in the in-phase condition in the high frequency ranges.

The simple effect test for finger combination × phase mode × frequency interaction revealed significant simple-simple main effects in *φ*_*I*_ in the frequency ranges 5–10, 10–15, 15–20, 20–25 and 25–30 s, and significant difference between the in-phase and anti-phase conditions (F(1,9) = 4.654, *p*< .05, F(1,9) = 5.770, *p*< .05, F(1,9) = 65.501, *p*< .001, F(1,9) = 41.255, *p*< .001, F(1,9) = 8.807, *p*< .005, respectively). The results correspond with the result of the above SD φ analysis comparing the four-finger condition with the two-finger condition. It also revealed a significant simple-simple main effect in *φ*_*M*_ in the frequency ranges 10–15, 15–20, 20–25 and 25–30 s, and significant difference between the in-phase and anti-phase conditions (F(1,9) = 4.265, *p*< .05, F(1,9) = 5.930, *p*< .05, F(1,9) = 31.857, *p*< .001, F(1,9) = 34.123, *p*< .001, respectively). In summary, SD φ of between-hand (*φ*_*I*_ and *φ*_*M*_) became larger in the anti-phase condition than in the in-phase condition in the high frequency ranges. However, no significant difference was found in SD φ of within-hand (*φ*_*L*_ and *φ*_*R*_) even in the high frequency ranges.

#### Cross recurrence quantification analysis

A three-way ANOVA (finger combination (4) × phase mode (2) × frequency (6)) conducted on %*REC* confirmed the main effect of the finger combination (F(1,9) = 4.066, *p*< .05), phase mode (F(1,9) = 10.006, *p*< .05), and frequency (F(1,9) = 7.930, *p*< .001). It also revealed significant interactions: finger combination × phase mode (F(1,9) = 15.137, *p*< .001) and finger combination × frequency (F(1,9) = 2.356, *p*< .005).

The simple main effect test for finger combination × phase mode interaction revealed significant difference in combination of index and middle fingers (%*REC*_*I*_ and %*REC*_*M*_) and within right hand (%*REC*_*R*_) between the in-phase and anti-phase conditions (F(1,9) = 25.781, *p*< .001, F(1,9) = 11.553, *p*< .005, F(1,9) = 5.072, *p*< .05, respectively). %*REC* was significantly higher in the in-phase mode than in the anti-phase mode in these finger combinations.

The simple main effect test for finger combination × frequency interaction revealed significant difference in the frequency range 0–5, 5–10 and 20–25 s among finger combinations (F(1,9) = 8.462, *p*< .001, F(1,9) = 3.747, *p*< .05, F(1,9) = 3.535, *p*< .05, respectively). %*REC* varied among finger combinations in these frequency ranges, but no common pattern of the differences was found.

A three-way ANOVA (finger combination (4) × phase mode (2) × frequency (6)) conducted on *L* confirmed the main effect of finger combination (F(1,9) = 7.840, *p*< .001), phase mode (F(1,9) = 7.869, *p*< .05), and frequency (F(1,9) = 31.595, *p*< .001). It also revealed significant interactions: finger combination × phase mode (F(1,9) = 10.718, *p*< .001), finger combination × frequency (F(1,9) = 2.438, *p*< .005).

The simple main effect test for finger combination × phase mode interaction revealed significant difference in combination of index and middle fingers (*L*_*I*_ and *L*_*M*_) between the in-phase and anti-phase conditions (F(1,9) = 24.358, *p*< .001, F(1,9) = 9.023, *p*< .005, respectively). However, it revealed no significant difference in the combination of left and right hands (*L*_*L*_ and *L*_*R*_) between the in-phase and anti-phase conditions (F(1,9) = 0.101, *p* = .7519, N.S., F(1,9) = 1.356, *p* = .2520, N.S., respectively). In summary, although in the case of between-hand combination (*L*_*I*_ and *L*_*M*_) *L* was significantly longer in the in-phase mode than in the anti-phase mode, no significant difference was found in *L* between two phase modes in the case of within-hand combinations (*L*_*L*_ and *L*_*R*_).

The simple main effect test for finger combination × frequency interaction revealed significant difference in combination of index fingers (*L*_*I*_) in most frequency ranges 0–5, 10–15, 15–20, 20–25 and 25–30 s (F(1,9) = 5.297, *p*< .005, F(1,9) = 3.193, *p*< .05, F(1,9) = 3.689, *p*< .05, F(1,9) = 15.412, *p*< .001, F(1,9) = 3.212, *p*< .05, respectively). *L* varied among finger combinations across a trial, but no common pattern of the differences was found.

In summary, results of additional analyses on the four-finger condition suggested as the common finding that the movement stability was significantly higher in the in-phase condition than in the anti-phase condition in the between-hand combinations. In the within-hand combinations, however, the movement stability did not significantly differ between two phase modes with exception of %*REC*_*R*_.

## Discussion

### Two-finger condition

Results of Experiment 1 show that, in the two-finger condition, all measures of the movement stability (i.e., the percentage of phase transition occurrence, *SD φ*_*I*_, %*REC*, *L*) did not differ significantly between the in-phase and anti-phase modes. These results differ from the prediction from the HKB model [4] (that is, the movement stability is higher in the in-phase condition than in the anti-phase condition at high frequency). A comparison in the anti-phase condition between the two-finger and four-finger conditions revealed that the movement stability (*SD φ*_*I*_, %*REC*, *L*) was higher in the two-finger condition than in the four-finger condition. A comparison in the in-phase condition between the two-finger and four-finger conditions also revealed that the movement stability (%*REC*, *L*) was higher in the two-finger condition than in the four-finger condition, or equally stable between them in the case of SD *φ*_*I*_. From these facts, it was inferred that the stabilization of the two-finger anti-phase condition may bring the result.

**Fig 9:**
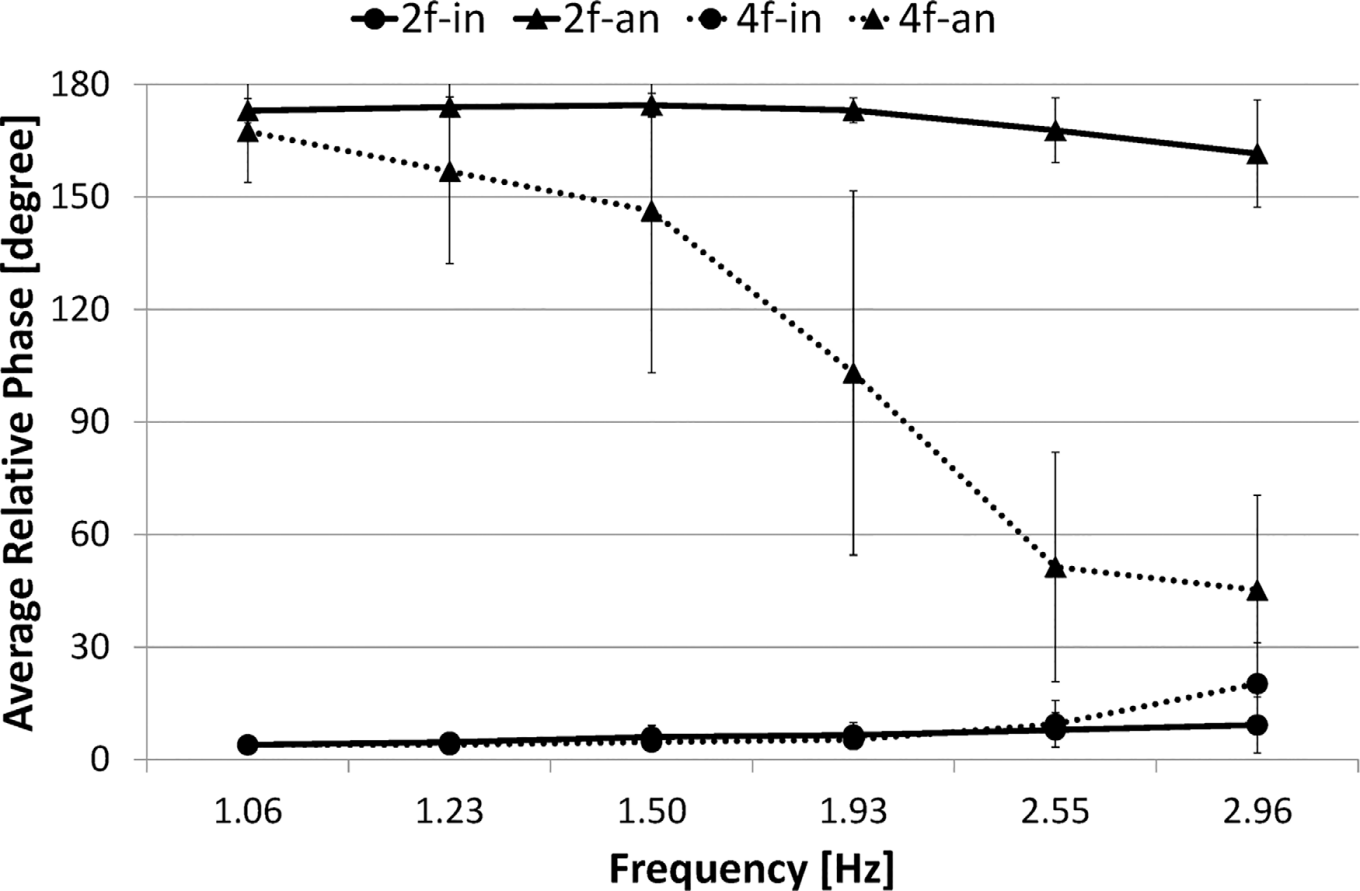
Average Relative Phase Distribution. Circle marker/rigid line represents two-finger in-phase condition, 2f-in; Triangle marker/rigid line represents two-finger anti-phase condition, 2f-an; Circle marker/dashed line represents four-finger in-phase condition, 4f-in; Triangle marker/dashed line represents four-finger anti-phase condition, 4f-an. Error bars represent the standard deviation.

One might presume that anti-phase was the only pattern maintained at high frequency in the anti-phase trial. The possibility of that, however, can be denied by the analysis of the percentage of phase transition occurrence: If in the anti-phase trial the initial phase mode (i.e., anti-phase) changes to in-phase mode. Then the analysis detects it and counts it as a phase transition. In addition, to confirm whether any other phase mode exists, the average relative phase distribution (of all trials) was shown for the two-finger anti-phase condition (Fig 9). Each plot corresponds to the absolute values of relative phase averaged across each frequency range (5 s duration) shown. Accordingly, it was confirmed that anti-phase was the only pattern maintained even at high frequency in the two-finger anti-phase trial (i.e., 2f-an). Several factors leading to such a result are regarded as follows.

#### Frequency Effect

The first factor is the range of metronome frequency that controlled the movement frequency. In the current experiments, the frequency was controlled from 1 Hz to 3 Hz, as in earlier studies [26, 27]. The critical frequency for finger movement is reportedly about 2.2 Hz [2, 18]. For that reason, the frequency range was presumed to cover the critical point. The present study did not examine higher frequencies because participants in our preliminary experiment were unable to perform the task at a higher frequency (5 Hz) across trials because of fatigue from moving the fingers so quickly and for such a long time. Therefore the present study used the frequency range of 1–3 Hz. However, reports of earlier studies [49, 50] that set the frequency higher than 3 Hz described that the in-phase mode was more stable than the anti-phase mode at high frequencies. These results may suggest that the metronome frequency used for this experiment did not cover the critical frequency. However, some differences exist between the experimental situations used for this study and those used in a previous study [49]. Although the metronome frequencies of the in-phase mode and anti-phase mode did not differ in this study, as shown in Fig 3 (i.e., one beat per cycle, single-metronome), the metronome frequencies used in the previous study [49] differed between modes (i.e., one beat per cycle in the in-phase condition, with two beats per cycle, double-metronome, in the anti-phase condition so that both left and right fingers can tap on the beat). In general, the movement is said to be more stable in the double-metronome condition than in the single-metronome condition by coupling between an external event (i.e., auditory metronome) and movement (see explanations of the *anchoring effect* [10, 51, 52]). Nevertheless, an earlier report [49] described that too much information can destabilize the movement. Actually, results of some previous studies suggest that the stabilizing effect of sensory information depends on several factors such as the kind of available sensory information, the combination of information, its phase relation, and its frequency [20]. Further investigations must be undertaken to ascertain which factors contributed to the result, and to what degree they did so.

#### Haptic Information

However, the critical frequency of a finger movement such as wiggling is generally about 2.2 Hz [2, 18]. Therefore, the reason why the anti-phase finger-tapping movement can maintain stability over the frequency must be examined. Regarding this point, we presume that, unlike other movement tasks such as finger wiggling, the finger-tapping task participants were required to tap on the desk surface. For that reason, haptic information was available at the time of the tapping. Kelso, Fink, DeLaplain, and Carson [20] reported that haptic information can stabilize finger extension-flexion movement. In reference to such a kind of factor, Loesby, Piek and Barrett [53] reported movement force can influence the bimanual finger-tapping patterns, and argued that force should be considered as a *control parameter*. Therefore, we infer that such haptic information can affect the stability of the anti-phase tapping movement.

### Four-finger condition

In the four-finger condition, all measures of the movement stability were significantly greater in the in-phase mode than in the anti-phase mode. This result agreed with the results of an earlier study of intrapersonal four-finger tapping, so that results can be explained in terms of perceptual spatial symmetry [26], or/and co-activation of homologous muscles [2]. Comparing the result of the four-finger condition with that of the two-finger condition, haptic information that stabilize the anti-phase mode in the two-finger condition could not overcome the strong coupling within hands in the four-finger anti-phase condition. Further examinations must be conducted to clarify these several factors’ effects on inter-limb coordination dynamics by means of careful experimental manipulation and control.

Additional analyses in the four-finger condition provided further insight into the relation between “within-hand” coordination and “between-hand” coordination. Most measures of the movement stability (without %*REC*_*R*_) did not differ significantly “within-hand” coordination even at high frequency, although for “between-hand” coordination, the in-phase mode was more stable than the anti-phase mode at high frequency. We interpret this result as that the coupling of “within-hand” coordination was stronger than that of “between-hand” coordination in the four-finger intrapersonal tapping task.

### Number of Fingers × Phase Mode × Frequency Interaction

The result of analysis on *SD φ*_*I*_ suggests the movement stability does not significantly differ between the in-phase mode and anti-phase mode in the two-finger condition even over the critical frequency, on the other hand, it is significantly higher in the in-phase mode than in the anti-phase mode in the four-finger condition at most frequency. We interpret this result as that both the in-phase and anti-phase modes are equally stable in the two-finger condition regardless of the movement frequency range 1–3 Hz.

As a result of Experiment 1, for bimanual finger-tapping, it can be suggested that haptic information in terms of touching the environmental surface might affect the dynamics of the intrapersonal coordination system. If further examination would confirm it, new term/parameter should be added to the model. Although haptic information can probably stabilize anti-phase tapping movements in the intrapersonal system, what about interpersonal systems that have no neural or mechanical linkage between limbs? Intrapersonal coordination systems can be organized through perceptual and neuromuscular couplings. Interpersonal coordination system, however, is difficult to organize through neuromuscular couplings. It is important to investigate these two systems, which have different manners of coupling. At the same time, it is also challenging to reveal whether and how much the same principle of self-organization governs these two systems.

## Experiment 2: Interpersonal Tapping Experiment

### Method

For Experiment 2, a pair sat side-by-side. Exactly as in Experiment 1, four conditions were used: two-finger in-phase, two-finger anti-phase, four-finger in-phase, and four-finger anti-phase (Fig 1).

### Participants

Ten pairs of participants (5 pairs of men, 5 pairs of women; 20 healthy right-handed participants) participated in Experiment 2. Participants were recruited by distributing flyers to advertise the study or by sending e-mail. Participants included undergraduate students of other universities and business people as well as graduate students of the Institute. Pairs of participants were mutually acquaintance. All were 21–47 years old (average = 27.8). All participants had normal hearing and normal vision. The procedures were approved by the research ethics committee of the National Institute of Informatics, where the experiment was conducted. Each participant provided written informed consent to participate in this study. Each was paid 1,000 JPN yen/hr for their participation.

### Apparatus

**Fig 10:**
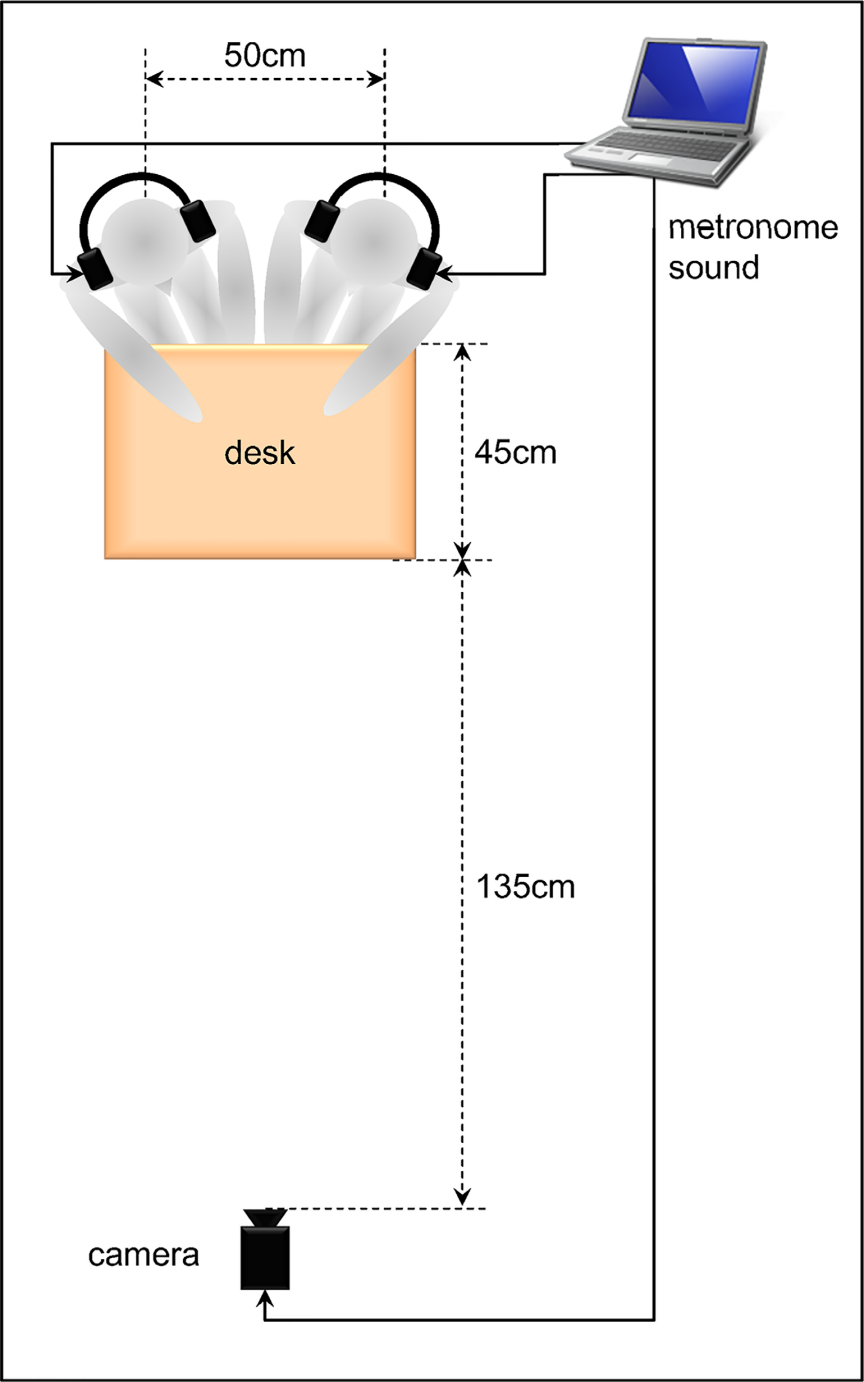
Experimental setup of Experiment 2. Experimental setup of an interpersonal experiment.

The same apparatus as that used in Experiment 1 was used in Experiment 2. The same metronome was presented to both participants through the individual headphones. Pairs of participants were seated about 50 cm apart from each other as shown in Fig 10.

### Design and Procedure

The same two factors were examined as those in Experiment 1: The phase mode, either in-phase or anti-phase, and the number of fingers, either two-finger or four-finger (Fig 1). Pairs of participants performed tasks in four conditions. Each condition was repeated four times. The order of the trials was determined randomly. Participants used only one hand, the outside hand, in Experiment 2. They were required to use the outside hand to control perceptual spatial symmetry factor [26, 54]. Using the outside hands in the interpersonal experiment, the perceptual spatial situation can be close to the situation using bimanual hands in the intrapersonal experiment. The task was identical to that used in Experiment 1.

### Data Analysis

Exactly as in Experiment 1, the percentage of phase transition occurrence, SD φ, the movement frequency and CRQA measures (%*REC* and *L*) for each condition were analyzed in Experiment 2.

## Results

### Coordination between index fingers

#### Percentage of phase transition occurrence

**Fig 11:**
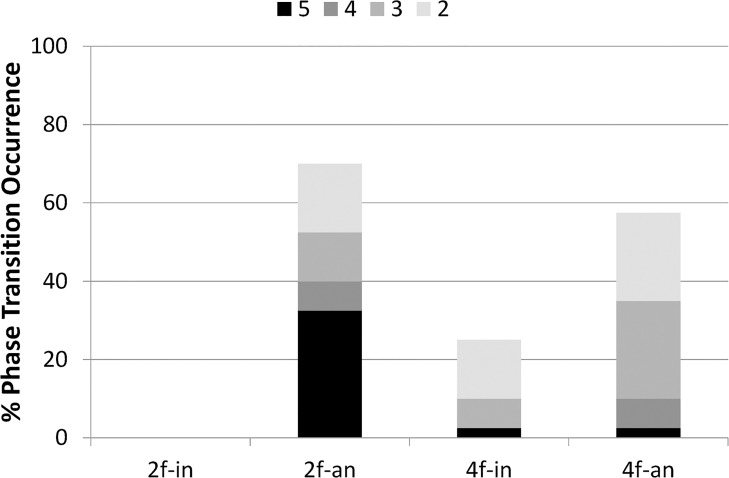
Percentage of phase transition occurrence. Colors of bar indicate how many times the taps in the opposite phase mode repeated. From black (5 times), … to the lightest gray (2 times). 2f-in: two-finger in-phase condition, 2f-an: two-finger anti-phase condition, 4f-in: four-finger in-phase condition, 4f-an: four-finger anti-phase condition.

Fig 11 portrays the percentage of phase transition occurrence for each condition (the number of how many times the taps in the opposite phase mode is 5). In the two-finger condition, no transition was observed in the in-phase condition, although it was observed at 32.5% in the anti-phase condition. In the four-finger condition, the transition occurred at 2.5% both in the in-phase and anti-phase condition.

A two-way ANOVA (number of fingers (2) × phase mode (2)) conducted on the percentage of phase transition occurrence revealed the main effect of the number of fingers (F(1,9) = 8.442, *p*< .05) and phase mode (F(1,9) = 18.778, *p*<.005). It also revealed significant interactions: number of fingers × phase mode (F = (1,9) = 9.447, *p*<.05).

The simple main effect test for number of fingers × phase mode interaction revealed a significant difference between the two-finger and four-finger conditions in the anti-phase condition (F(1,9) = 17.876, *p*< .001). It also revealed a significant difference between the in-phase and anti-phase modes in the two-finger condition (F(1,9) = 25.140, *p*< .001), but no significant difference between the in-phase and anti-phase modes in the four-finger condition (F(1,9) = 0.000, *p* = 1.000, N.S.). The percentage of phase transition occurrence was significantly higher in the anti-phase condition than in the in-phase mode in the two-finger condition, whereas it did not significantly differ between two phase modes in the four-finger condition. Fig 11 also shows that the index, the percentage of phase transition occurrence, changed depending on the parameter, the number of taps in the opposite phase mode, as it changes from 2 to 5.

#### SD of relative phase

**Fig 12:**
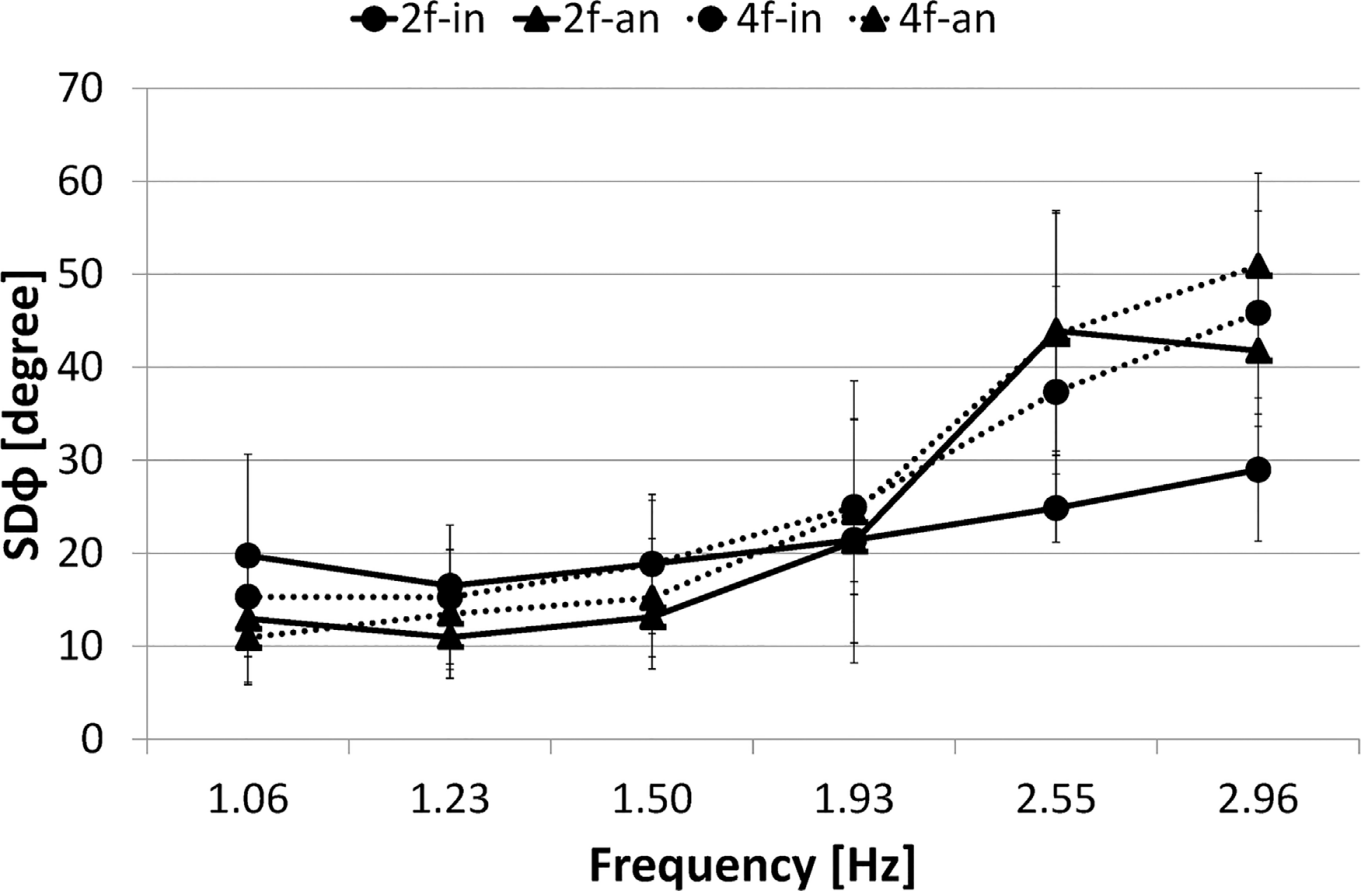
SD of relative phase. Circle marker/rigid line represents two-finger in-phase condition, 2f-in; Triangle marker/rigid line represents two-finger anti-phase condition, 2f-an; Circle marker/dashed line represents four-finger in-phase condition, 4f-in; Triangle marker/dashed line represents four-finger anti-phase condition, 4f-an. Error bars represent the standard deviation.

Fig 12 presents the standard deviation of the relative phase between index fingers (*φ*_*I*_) as a function of elapsed time of the trial (grouped into six 5 s durations), which is equivalent to the frequency of movement.

A three-way ANOVA (number of fingers (2) × phase mode (2) × frequency (6)) conducted on the SD *φ*_*I*_ confirmed the main effect of number of fingers (F(1,9) = 15.964, *p*< .005) and frequency (F(1,9) = 110.536, *p*< .001). It also revealed significant interactions: number of fingers × frequency (F(1,9) = 6.627, *p*< .001), phase mode × frequency (F(1,9) = 11.302, *p*< .001) and number of fingers × phase mode × frequency (F(1,9) = 3.587, *p*< .01).

The simple main effect test for number of fingers × frequency interaction revealed significant difference in the high frequency ranges (20–25 and 25–30 s) between two-finger and four-finger conditions (F(1,9) = 7.818, *p*< .01, F(1,9) = 36.015, *p*< .001, respectively). SD *φ*_*I*_ was significantly larger in the four-finger condition than in the two-finger condition in the high frequency range.

The simple main effect test for phase mode × frequency interaction revealed significant differences in the frequency ranges 0–5, 10–15, 20–25 and 25–30 s between the in-phase and anti-phase conditions (F(1,9) = 6.009, *p*< .05, F(1,9) = 4.204, *p*< .05, F(1,9) = 30.541, *p*< .001, F(1,9) = 15.226, *p*< .001, respectively). SD *φ*_*I*_ was significantly larger in the anti-phase condition than in the in-phase condition in the specific frequency ranges, but no pattern in its differences was found specifically.

The simple effect test for number of fingers × phase mode × frequency interaction revealed a significant simple-simple main effect in the two-finger condition in the frequency ranges 0–5, 10–15, 20–25, and 25–30 s, and significant difference between the in-phase and anti-phase conditions (F(1,9) = 5.597, *p*< .05, F(1,9) = 3.993, *p*< .05, F(1,9) = 44.054, *p*< .001, F(1,9) = 19.879, *p*< .001, respectively). It also revealed significant simple-simple main effect in the four-finger condition in the frequency range 20–25 s, and significant difference between the in-phase and anti-phase conditions (F(1,9) = 4.666, *p*< .05). In summary, SD *φ*_*I*_ was significantly larger in the anti-phase condition than in the in-phase condition in the two-finger condition, especially in the high frequency ranges. Whereas in the four-finger condition only in the specific frequency range (20–25 s) SD *φ*_*I*_ was significantly larger in the anti-phase condition than the in-phase condition. No significant difference was found between the two phase modes in the other frequency ranges.

#### Cross recurrence quantification analysis

**Fig 13:**
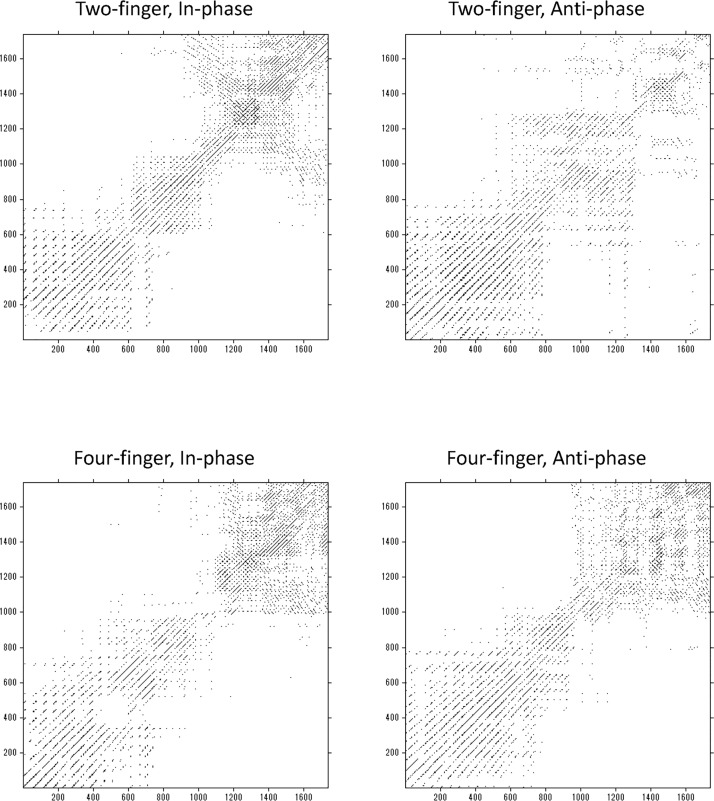
Cross recurrence plots. Cross recurrence plots of sample data for each of four conditions. Left-top, two-finger in-phase condition; Right-top, two-finger anti-phase condition; Left-bottom, four-finger in-phase condition; Right-bottom, four-finger anti-phase condition.

**Fig 14:**
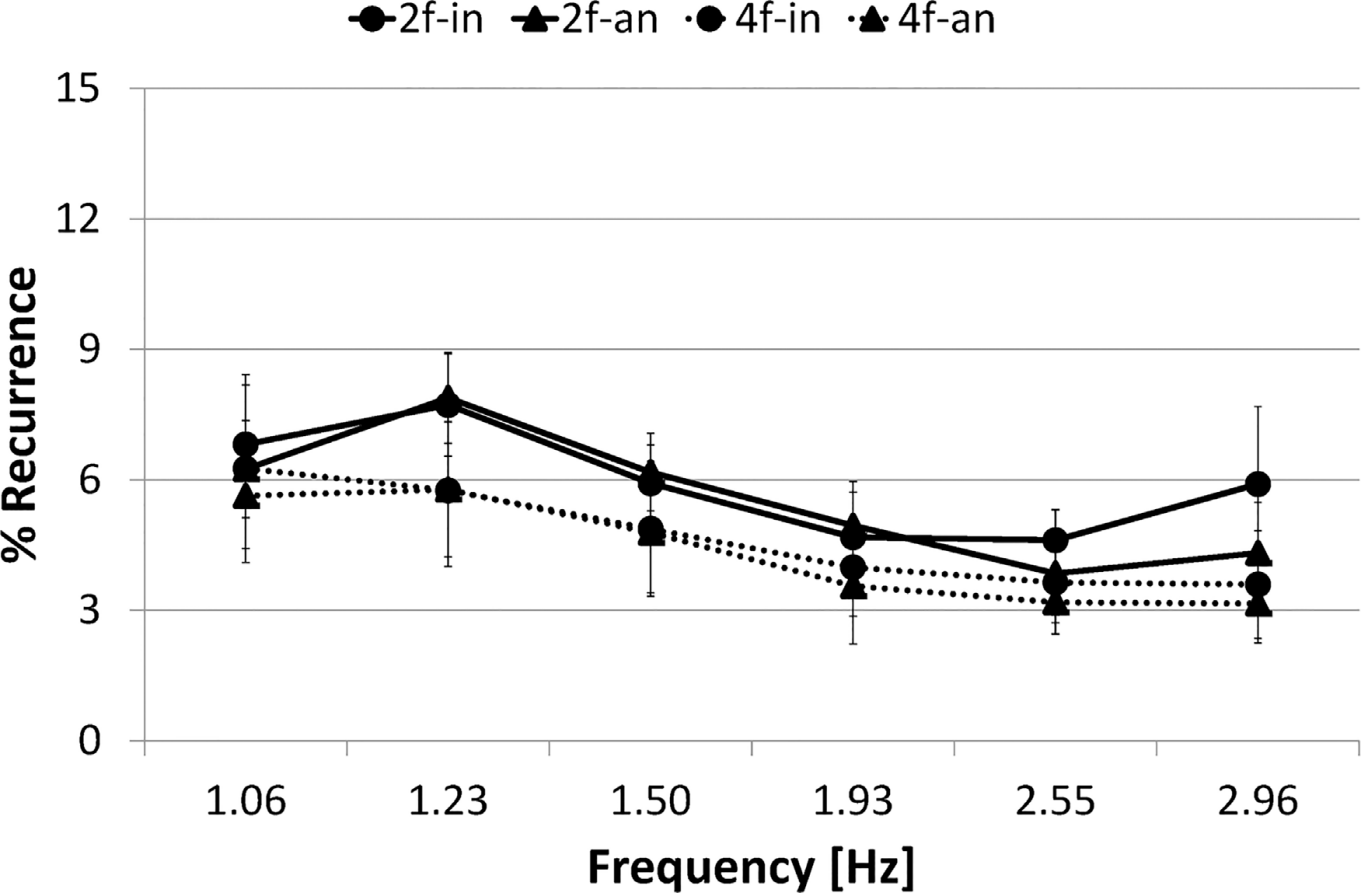
%Recurrence. Circle marker/rigid line represents two-finger in-phase condition, 2f-in; Triangle marker/rigid line represents two-finger anti-phase condition, 2f-an; Circle marker/dashed line represents four-finger in-phase condition, 4f-in; Triangle marker/dashed line represents four-finger anti-phase condition, 4f-an. Error bars represent the standard deviation.

Fig 13 represents cross recurrence plots of sample data for each of four conditions and Fig 14 presents %*REC*_*I*_ for each condition as a function of frequency.

A three-way ANOVA (number of fingers (2) × phase mode (2) × frequency (6)) conducted on %*REC*_*I*_ confirmed the main effect of number of fingers (F(1,9) = 18.109, *p*< .005), phase mode (F(1,9) = 14.894, *p*< .005) and frequency (F(1,9) = 33.652, *p*< .001). It also revealed significant interactions: number of fingers × frequency (F(1,9) = 5.142, *p*< .001), phase mode × frequency (F(1,9) = 3.201, *p*< .05), and number of fingers × phase mode × frequency (F(1,9) = 2.726, *p*< .05).

The simple main effect test for number of fingers × frequency interaction revealed significant difference in the frequency ranges 5–10, 10–15, 15–20, 20–25, and 25–30 s between the two-finger and four-finger conditions (F(1,9) = 30.839, *p*< .001, F(1,9) = 11.044, *p*< .005, F(1,9) = 8.064, *p*< .01, F(1,9) = 4.999, *p*< .05, F(1,9) = 22.488, *p*< .001, respectively). %*REC*_*I*_ was significantly higher in the two-finger condition than in the four-finger condition in most frequency ranges, with the exception of the first range of 0–5 s.

The simple effect test for phase mode × frequency interaction revealed a significant simple-simple main effect in the frequency ranges 0–5, 20–25 and 25–30 s, and significant difference between the in-phase and anti-phase conditions (F(1,9) = 5.811, *p*< .05, F(1,9) = 6.063, *p*< .05, F(1,9) = 16.562, *p*< .001, respectively). Results show that %*REC*_*I*_ became significantly higher in the in-phase condition than in the anti-phase condition in the lowest frequency range 0–5 s and higher frequency ranges 20–30 s.

The simple effect test for number of fingers × phase mode × frequency interaction revealed significant difference between the in-phase and anti-phase conditions in the two-finger condition in the high frequency ranges 20–25 and 25–30 s (F(1,9) = 5.783, *p*< .05, F(1,9) = 24.756, *p*< .001, respectively), but no significant difference between two phase modes in the four-finger condition in all frequency ranges 0–5, 5–10, 10–15, 15–20, 20–25 and 25–30 s (F(1,9) = 3.904, *p* = .0507, N.S., F(1,9) = 0.004, *p* = .9517, N.S., F(1,9) = 0.073, *p* = .7873, N.S., F(1,9) = 1.828, *p* = .1792, N.S., F(1,9) = 2.073, *p* = .1528, N.S., F(1,9) = 1.901, *p* = .1708, N.S., respectively). In summary, in the two-finger condition, %*REC*_*I*_ was significantly higher in the in-phase mode than in the anti-phase mode at high frequency. On the other hand, in the four-finger condition, %*REC*_*I*_ did not significantly differ between two phase modes across a trial.

**Fig 15:**
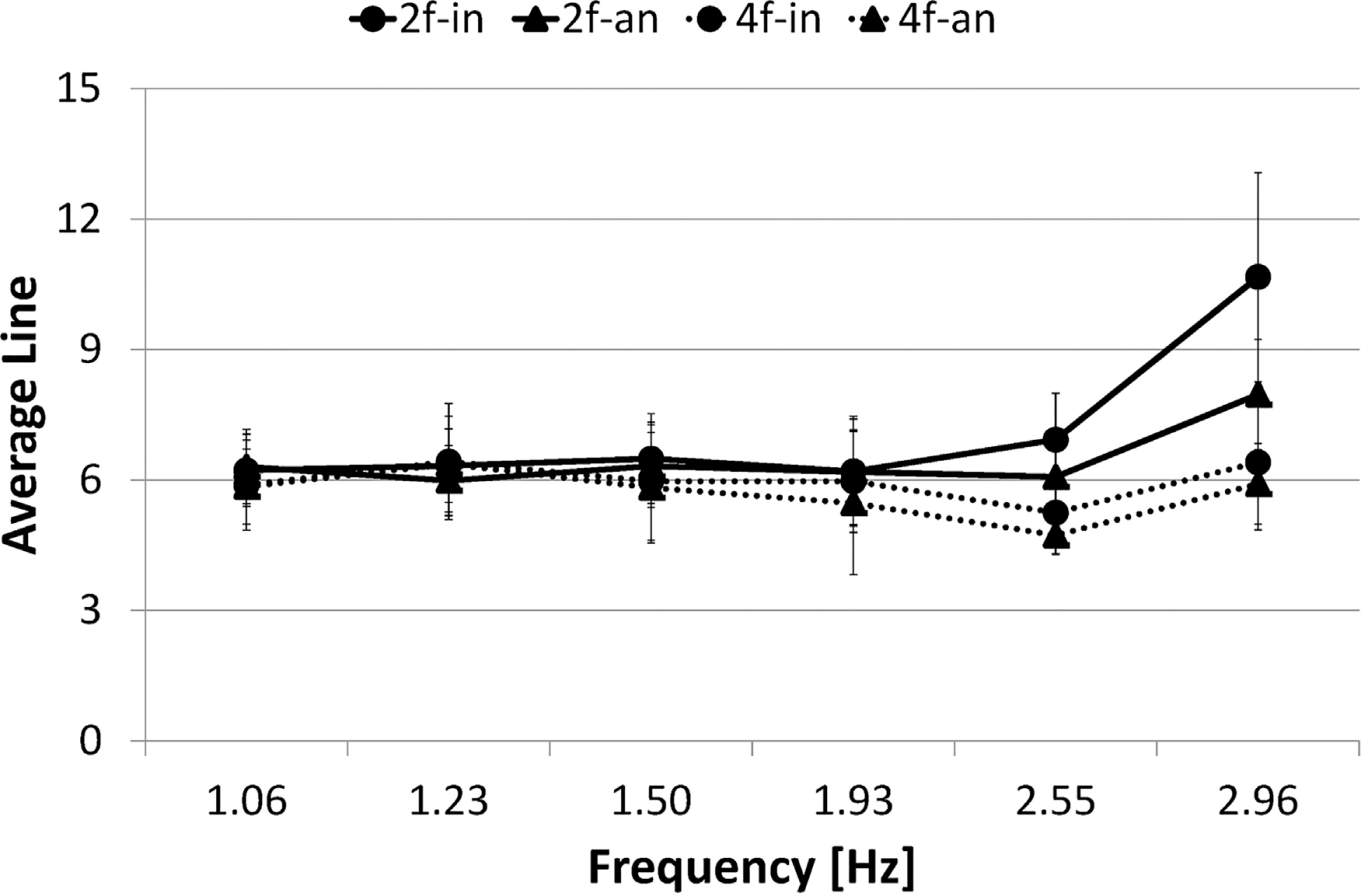
Average line length. Circle marker/rigid line represents two-finger in-phase condition, 2f-in; Triangle marker/rigid line represents two-finger anti-phase condition, 2f-an; Circle marker/dashed line represents four-finger in-phase condition, 4f-in; Triangle marker/dashed line represents four-finger anti-phase condition, 4f-an. Error bars represent the standard deviation.

Fig 15 presents *L*_*I*_ for each condition as a function of frequency. A three-way ANOVA (number of fingers (2) × phase mode (2) × frequency (6)) conducted on *L*_*I*_ confirmed the main effects of the number of fingers (F(1,9) = 14.115, *p*< .005), phase mode (F(1,9) = 18.257, *p*< .005) and frequency (F(1,9) = 12.420, *p*< .001). It also revealed significant interactions: number of fingers × frequency (F(1,9) = 20.598, *p*< .001), phase mode × frequency (F(1,9) = 5.472, *p*< .001), and number of fingers × phase mode × frequency (F(1,9) = 8.414, *p*< .001).

The simple main effect test for number of fingers × frequency interaction revealed significant difference in the frequency ranges 20–25, and 25–30 s between the two-finger and four-finger conditions (F(1,9) = 18.157, *p*< .001, F(1,9) = 79.332, *p*< .001, respectively). *L*_*I*_ was longer in the two-finger condition than in the four-finger condition in the high frequency ranges (20–30 s).

The simple main effect test for phase mode × frequency interaction revealed significant differences in the frequency ranges 20–25 and 25–30 s between the in-phase and anti-phase conditions (F(1,9) = 7.082, *p*< .05, F(1,9) = 38.279, *p*< .001, respectively). *L*_*I*_ was significantly longer in the in-phase condition than in the anti-phase condition in the high frequency ranges (20–30 s).

The simple effect test for number of fingers × phase mode × frequency interaction revealed significant simple-simple main effect in the two-finger condition in the frequency ranges 20–25 and 25–30 s, and significant difference between the in-phase and anti-phase conditions (F(1,9) = 7.486, *p*< .01, F(1,9) = 74.458, *p*< .001, respectively), but no significant difference between two phase modes in the four-finger condition in all frequency ranges 0–5, 5–10, 10–15, 15–20, 20–25 and 25–30 s (F(1,9) = 0.015, *p* = .9040, N.S., F(1,9) = 0.033, *p* = .8562, N.S., F(1,9) = 0.220, *p* = .6396, N.S., F(1,9) = 2.572, *p* = .1117, N.S., F(1,9) = 2.759, *p* = .0996, N.S., F(1,9) = 2.540, *p* = .1139, N.S., respectively). In summary, in the two-finger condition, *L*_*I*_ was significantly longer in the in-phase condition than in the anti-phase condition in the high frequency ranges, but in the four-finger condition, no significance was found in *L* between two phase modes over a trial.

Results of analyzing the movement frequency revealed that the average maximum frequency across trials in which phase transition occurred was 2.929 Hz (SD = 0.167), and the average maximum frequency across trials in which no phase transition occurred was 2.924 Hz (SD = 0.101). Results of Welch’s *t*-test indicated no significant difference between the average maximum frequencies of the two groups (i.e., the phase-transition group and the no phase transition group) (*t*(21) = 0.168, *p* = 0.872, N.S.).

### Additional analyses of four-finger condition

In four fingers condition, the same additional analyses as Experiment 1 on the standard deviation of the finger combination in four finger combinations (*φ*_*I*_, *φ*_*M*_, *φ*_*L*_, *φ*_*R*_) were conducted. CRQA also was conducted on four finger combinations, and two measures (%*REC* and *L*) were obtained for each finger combination, i.e., between index fingers of pairs of participants (%*REC*_*I*_, *MAXL*_*I*_), between the middle fingers of pairs of participants (%*REC*_*M*_, *MAXL*_*M*_), between index and middle fingers of the left participant (%*REC*_*L*_, *MAXL*_*L*_), and the right participant (%*REC*_*R*_, *MAXL*_*R*_).

#### SD of relative phase

A three-way ANOVA (finger combination (4) × phase mode (2) × frequency (6)) conducted on the SD of four finger combinations (i.e., *φ*_*I*_, *φ*_*M*_, *φ*_*L*_, *φ*_*R*_), confirmed the main effect of finger combination (F(1,9) = 52.742, *p*< .001) and frequency (F(1,9) = 135.894, *p*< .001). It also revealed significant interactions: finger combination × frequency (F(1,9) = 17.882, *p*< .001) and finger combination × phase mode × frequency (F(1,9) = 3.010, *p*< .001).

Results of multiple comparisons using Ryan’s method [48] in the main effect of finger combination revealed a significant difference between *φ*_*M*_ and *φ*_*R*_ (t(27) = 9.243, *p*< .001), between *φ*_*M*_ and *φ*_*L*_ (t(27) = 8.820, *p*< .001), between *φ*_*I*_ and *φ*_*R*_ (t(27) = 8.954, *p*< .001) and between *φ*_*I*_ and *φ*_*L*_ (t(27) = 8.531, *p*< .001). In summary, SD φ was larger in the between-individual case (*φ*_*I*_ and *φ*_*M*_) than in the within-individual case (*φ*_*L*_ and *φ*_*R*_).

The simple main effect test for finger combination × frequency interaction revealed significant differences in the frequency ranges 0–5, 10–15, 15–20, 20–25 and 25–30 s among finger combination (F(1,9) = 4.487, *p*< .005, F(1,9) = 5.236, *p*< .005, F(1,9) = 9.537, *p*< .001, F(1,9) = 50.015, *p*< .001, F(1,9) = 96.307, *p*< .001, respectively). SD φ varied among finger combinations in most frequency ranges without 5–10 s, although no common pattern in its differences was found specifically.

The simple effect test for finger combination × phase mode × frequency interaction revealed significant simple-simple main effects in *φ*_*I*_ in the frequency ranges 20–25 and 25–30 s, and significant differences were found between the in-phase and anti-phase conditions (F(1,9) = 6.094, *p*< .05, F(1,9) = 4.051, *p*< .05, respectively). It also revealed significant simple-simple main effects in *φ*_*M*_ in the frequency range 20–25 s, and significant difference between the in-phase and anti-phase conditions (F(1,9) = 13.001, *p*< .001). SD φ of between individuals (*φ*_*I*_ and *φ*_*M*_) varied in the high frequency ranges, although no common pattern in its differences was found specifically.

#### Cross recurrence quantification analysis

A three-way ANOVA (finger combination (4) × phase mode (2) × frequency (6)) conducted on %*REC* confirmed the main effect of frequency (F(1,9) = 16.831, *p*< .001).

A three-way ANOVA (finger combination (4) × phase mode (2) × frequency (6)) conducted on *L* confirmed the main effect of frequency (F(1,9) = 85.867, *p*< .001).

As a result of additional analyses of the four-finger condition, SD φ was larger in the between-individual case (*φ*_*I*_ and *φ*_*M*_) than in the within-individual case (*φ*_*L*_ and *φ*_*R*_).

## Discussion

### Two-finger condition

In the two finger condition, the percentage of phase transition occurrence and *SD φ*_*I*_ was significantly greater in the anti-phase mode than in the in-phase mode in the high frequency ranges. %*REC* and *L* were significantly greater in the in-phase mode than in the anti-phase mode in the high frequency ranges. These results demonstrated that the movement stability was higher in the in-phase mode than in the anti-phase mode specifically at high frequency. These results show good agreement with results obtained in a previous study [10]. The visual information might involve the organization and stabilization of coordinated finger-tapping movements. The auditory information, in terms of the sound participants’ tapping, was not available because of wearing a noise-canceling headphone. No participant reported that they could hear the sound. Moreover, as reported from an earlier study [10], one participant in the interpersonal tapping experiment was always able to perceive the metronome beats as auditory information, or an on-the-beat situation. However, the other participant must always tap at the midpoint between the metronome beats: an off-the-beat situation. This asymmetric situation of the participants should be considered because the off-the-beat participant might not couple with visual information, i.e., the partner’s finger’s motion, but rather auditory information, i.e., the metronome beats (see [34]). Additional experiments examining control of such an asymmetry effect must be conducted to ascertain which kind of perceptual information, visual or auditory, is involved in the organization and stabilization of interpersonal coordination systems.

### Four-finger condition

In the four-finger condition, the percentage of phase transition occurrence and SD *φ*_*I*_ (in most frequency ranges without 20–25 s), %*REC* and *L* did not differ significantly between the two phase modes, which suggests that no significant difference exists between the two phase modes in terms of the movement stability. These results differ from the prediction from the previous study [4, 10] (that is, the movement stability is higher in the in-phase condition than in the anti-phase condition at high frequency). A comparison in the in-phase condition between the two-finger condition and four-finger conditions revealed that the movement stability (SD *φ*_*I*_, %*REC* and *L*) was higher in the two-finger condition than in the four-finger condition at high frequency. A comparison in the anti-phase condition between the two-finger condition and four-finger conditions also revealed that the movement stability (SD *φ*_*I*_, %*REC* and *L*) was higher in the two-finger condition than in the four-finger condition at high frequency. From these facts, it was inferred that the loss of stability in the four-finger in-phase condition at high frequency led to the result. Why then did the four-finger in-phase condition become unstable at high frequency?

#### Complexity in Interactions between Oscillators

We discuss this point next in terms of complexity in the interaction between limbs derived from the multi-limb nested structure. In Experiment 2, participants were asked to coordinate their movement with partners and their own two fingers (i.e., index and middle fingers) simultaneously in the four-finger condition. For such a nested structure consisting of two intrapersonal coordination systems, the four-finger condition is presumably more complicated and difficult for participants to perform the task than the two-finger condition in terms not only of neural and mechanical coupling between oscillators but also of perceptual and attentional resources.

In such a complex nested system, participants must perform a kind of dual task in a simultaneous choice situation. In a nested system, one component (e.g., index finger of right participant) belongs simultaneously to the intrapersonal level (i.e., system consisting of index and middle fingers of the right participant) and to the interpersonal level (i.e., system consisting of index and middle fingers of right and left participants). The dynamics of both the intrapersonal and interpersonal levels might simultaneously be mutually influential if error or noise arises in one component (e.g., index finger of right participant). At this moment, if another component (e.g., middle finger of left participant) attempts to compensate for the error and maintain the stability of systems, the attempt can only succeed at either an intrapersonal or interpersonal level. Therefore, it is necessary to choose the stabilization of either the intrapersonal or interpersonal level. Here it does not mean a cognitive mechanism. The system is subject to the restriction of choice between the two from two levels (such that either of the two should be selected). If the coupling strength of the intrapersonal level is stronger than that of the interpersonal level, then the present result can be understood. Actually as a result of additional analyses in the four-finger condition, the movement stability (SD φ*ϕ*_I_) is higher in the “within-individual” (intrapersonal) coordination than in the “between-individual” (interpersonal) coordination.

Some researchers have addressed such interaction between intrapersonal and interpersonal coordination systems [28–31, 55]. Coey, Varlet, Schmidt, and Richardson [28] attempted to compare these two systems and to evaluate the relation between the stability of intrapersonal coordination and the emergence of spontaneous interpersonal coordination in pendulum-swinging experiments. Their experiments revealed that the stability of intrapersonal coordination and the emergence of interpersonal coordination were mutually independent [28]. Additional empirical studies and theoretical investigations are expected to be conducted to clarify the coordination dynamics of such a nested system and its complexity in the interaction between intrapersonal and interpersonal levels.

### Number of Fingers × Phase Mode × Frequency Interaction

The result of analysis on SD φ, %*REC* and *L* suggests the movement stability does not significantly differ between the in-phase mode and anti-phase mode in the four-finger condition even over the critical frequency. On the other hand, it is significantly higher in the in-phase mode than in the anti-phase mode in the two-finger condition at high frequency. We interpret this result as that both the in-phase and anti-phase modes are equally unstable in the four-finger condition regardless of the movement frequency range 1–3 Hz.

As a result of Experiment 2, for bimanual finger-tapping, it can be suggested that the complexity in the interactions between limbs, derived from multi-limbs and nested structure, might affect the dynamics of the interpersonal coordination system.

## General discussion

To summarize results obtained from Experiments 1 and 2, we first confirmed the difference in the connections among limbs between the intrapersonal and interpersonal coordination systems. The intrapersonal coordination system enables limbs to interact not only through perception (e.g., visual and haptic)-action coupling but also in mechanical and neuromuscular ways. However, the interpersonal coordination system enables its limbs to interact only through perception-action coupling by visually perceiving limb movement in the present experimental situation.

In Experiment 1 (intrapersonal experiment), results obtained under the four-finger conditions agreed with results predicted by the HKB model. However, results obtained under the two-finger conditions differed from predictions by the model. For results obtained in the two-finger condition, the stabilizing effect of haptic information was regarded as affecting the intrapersonal coordination system (Experiment 1), but not the interpersonal one (Experiment 2).

In Experiment 2 (interpersonal experiment), results obtained under the two-finger conditions agreed with the prediction. Results under the four-finger conditions differed from the prediction. Adding to the lack of haptic linkage, for the interpersonal coordination system that has a nested structure, the complexity in the interaction between the intrapersonal and interpersonal levels was regarded as the destabilizing factor for the in-phase tapping movement in the four-finger condition. Although the actual effects of such perceptual information and the complexity in the interaction remain unclear, the results reported herein suggest that these factors can engender the different coordination dynamics of the two systems. Comparison of results of the percentage of phase transition occurrence shows that the occurrence probability is robust in Experiment 1 (intrapersonal experiment), but it changes depending on the parameter that represents how many times tapping occurred in the opposite phase in Experiment 2 (interpersonal experiment). This result might derive from the difference in the nature of coupling between limbs. In an intrapersonal coordination system, once the transition occurs, the new pattern soon becomes stable. In contrast, with an interpersonal coordination system, even if the transition occurs, the new pattern does not become stable soon but the initial pattern appears to be retained.

In spite of careful experimentation and analysis, this study did not reject the null hypothesis in Experiment 1 (two-finger condition) or Experiment 2 (four-finger condition). Results of Experiment 1, demonstrate that haptic information might stabilize less stable and difficult finger movement, suggesting its application to improving bimanual dexterities in rehabilitation or learning new skills based on complex bimanual coordination such as that of playing the piano and typing on a keyboard. Results of Experiment 2 show that a nested structure of multi-oscillator systems might affect the coordination system’s stability and its dynamics. Further verification might improve understanding of coordinated behaviors between individuals. Although these results were negative results, they are worth reporting and are expected to motivate further comparison between intrapersonal and interpersonal coordination.

## Conclusions

For the present study, we conducted two experiments: intrapersonal and interpersonal tapping experiments. Although some results of two experiments agreed with the prediction by the HKB model, others failed to agree with the prediction. Results show that different dynamics were observed in intrapersonal and interpersonal coordination systems. For the intrapersonal coordination system it is suggested that haptic information may stabilize the anti-phase tapping movement in the two-finger condition. For the interpersonal coordination system it is suggested that the complexity in the interactions between limbs derived from multi-fingers and nested structure destabilize the in-phase tapping movement in the four-finger condition.

As described above, the HKB approach is useful and attractive because, using the same self-organizing principle, it can account for many situations, from individual-environment systems to interpersonal coordination systems. Results of the present studies are not contradictory to the model, but do not agree with the prediction from the model. They may suggest the addition of new term/parameter to the existing model.

## Supporting Information

S1 Dataset S1 is Supporting Information including Dataset used in analyses of Experiment 1. (XLSX)

S2 Dataset S2 is Supporting Information including Dataset used in analyses of Experiment 2. (XLSX)
